# Supplementary material for: Lentiviral gene therapy prevents anti-human acid α-glucosidase antibody formation in murine Pompe disease
Source: Mol Ther Methods Clin Dev. 2022 May 4;25:520–32. doi: 10.1016/j.omtm.2022.04.016 (PMC9127119; doi:10.1016/j.omtm.2022.04.016)
Supplement: Document S1. Figures S1 and S2 and Table S1 [file mmc1.pdf]

OMTM, Volume *25*

Supplemental information

Lentiviral gene therapy prevents anti-human acid α-glucosidase antibody formation in murine Pompe disease

Qiushi Liang, Eva C. Vlaar, Fabio Catalano, Joon M. Pijnenburg, Merel Stok, Yvette van Helsdingen, Arnold G. Vulto, Wendy W.J. Unger, Ans T. van der Ploeg, W.W.M. Pim Pijnappel, and Niek P. van Til

Table S1. Sequences of primers for qPCR

Primers	Sequence
HIV-U3 forward	5′-CTGGAAGGGCTAATTCACTC-3′
HIV-PSI reverse	5′-GGTTTCCCTTTCGCTTTCAG-3′
Sry forward	5′-TCATCGGAGGGCTAAAGTGTCAC-3′
Sry reverse	5′-TGGCATGTGGGTTCCTGTCC-3′
Gapdh forward	5′- TAATGGGGAGAGGTTCGATG -3′
Gapdh reverse	5′- GCTGCTTCCCGAGTAAAATG -3′

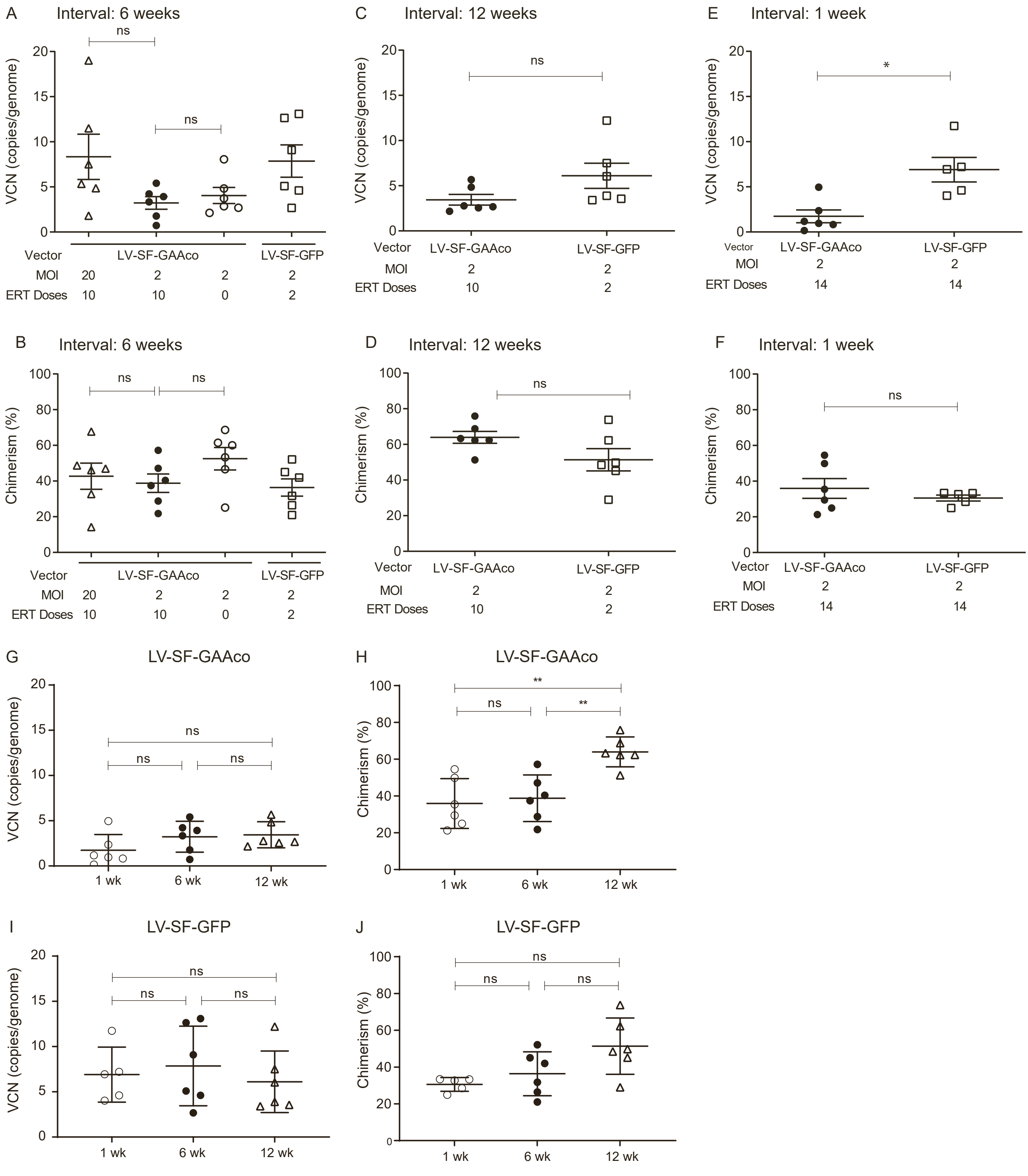

Fig. S1. Timing of immune tolerance induction: vector copy number and chimerism.

Vector copy number and chimerism in bone marrow. Graphs and symbols correspond to the experiments shown in Fig. 1. ERT was started 6 weeks (A, B), 12 weeks (C, D), or 1 week (E, F) after gene therapy. Side-by-side comparisons of vector copy number and chimerism in mice treated with LV-SF-GAA (G, H) and LV-SF-GFP (I, J). Bone marrow was collected at the end of the experiment and vector copy number and chimerism were determined by HIV and Sry qPCR, respectively, and normalized using Gapdh. Data are represented as means ± SEM; n=6 is all groups except in LV-SF-GFP treated mice in panels E and F (n=5). *p<0.05; ** p <0.01; ns, not significant.

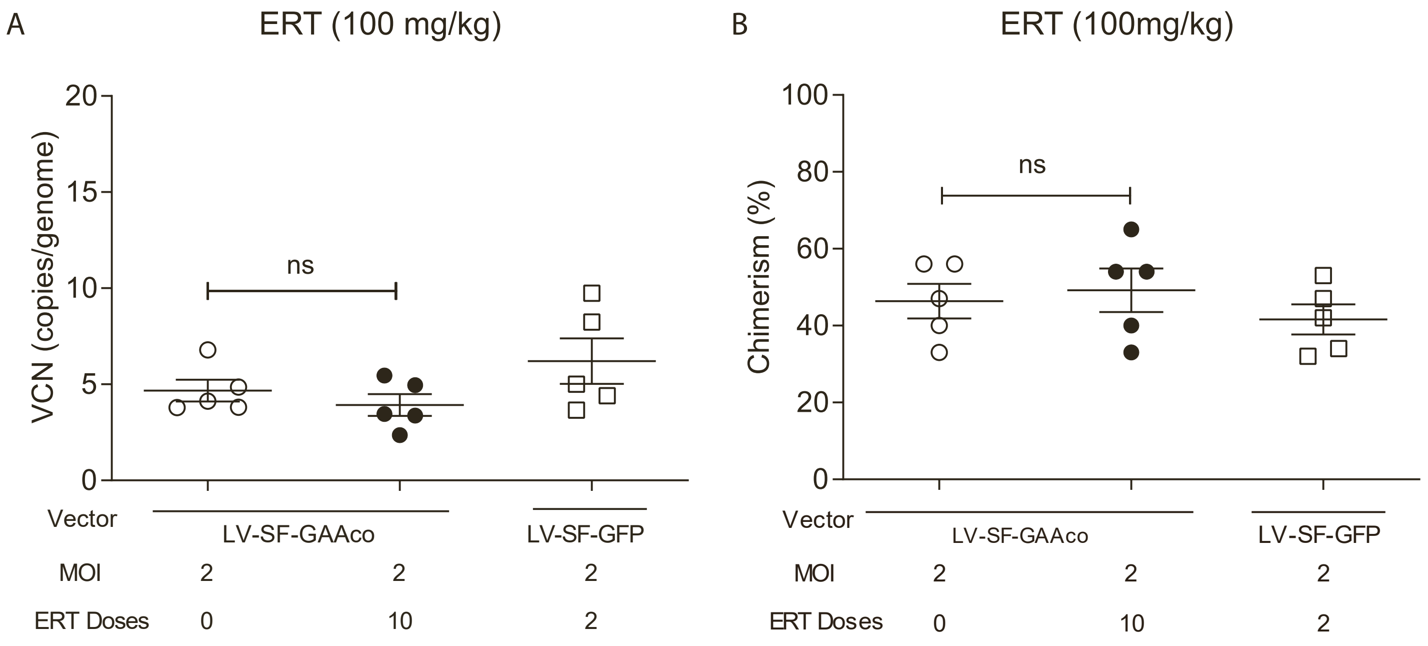

Fig. S2. Vector copy number and chimerism in mice receiving ERT doses at 100 mg/kg.
Graphs and symbols correspond to the experiment shown in Fig. 3. Vector copy number (A) and chimerism (B) were determined as described in in Fig. S1. Data are represented as means ± SEM; n=5 per group. Ns, not significant.
