# Supplementary material for: Lentiviral gene therapy prevents anti-human acid α-glucosidase antibody formation in murine Pompe disease
Source: Mol Ther Methods Clin Dev. 2022 May 4;25:520–32. doi: 10.1016/j.omtm.2022.04.016 (PMC9127119; doi:10.1016/j.omtm.2022.04.016)
Supplement: Document S2. Article plus supplemental information [file mmc2.pdf]

Molecular Therapy
Methods & Clinical Development
Original Article

Lentiviral gene therapy prevents anti-human acid α-glucosidase antibody formation in murine Pompe disease

Qiushi Liang,[1,2,3,4,9] Eva C. Vlaar,[2,3,4,9] Fabio Catalano,[2,3,4,9] Joon M. Pijnenburg,[2,3,4,10] Merel Stok,[2,3,4,5,10] Yvette van Helsdingen,[5] Arnold G. Vulto,[6] Wendy W.J. Unger,[7] Ans T. van der Ploeg,[3,4] W.W.M. Pim Pijnappel,[2,3,4,11] and Niek P. van Til[5,8,11]

[1]Department of Hematology and Research Laboratory of Hematology, West China Hospital, Sichuan University, Chengdu, Sichuan 610041, P.R. China; [2]Molecular Stem Cell Biology, Department of Clinical Genetics, Erasmus MC University Medical Center, 3015GE Rotterdam, the Netherlands; [3]Department of Pediatrics, Erasmus MC University Medical Center, 3015GE Rotterdam, the Netherlands; [4]Center for Lysosomal and Metabolic Diseases, Erasmus MC University Medical Center, 3015GE Rotterdam, the Netherlands; [5]Department of Hematology, Erasmus MC University Medical Center, 3015GE Rotterdam, the Netherlands; [6]Hospital Pharmacy, Erasmus MC University Medical Center, 3015GE Rotterdam, the Netherlands; [7]Laboratory of Pediatrics, Erasmus MC University Medical Center-Sophia Children's Hospital, 3015GE Rotterdam, the Netherlands

Enzyme replacement therapy (ERT) is the current standard treatment for Pompe disease, a lysosomal storage disorder caused by deficiency of the lysosomal enzyme acid alpha-glucosidase (GAA). ERT has shown to be lifesaving in patients with classic infantile Pompe disease. However, a major drawback is the development of neutralizing antibodies against ERT. Hematopoietic stem and progenitor cell-mediated lentiviral gene therapy (HSPC-LVGT) provides a novel, potential lifelong therapy with a single intervention and may induce immune tolerance. Here, we investigated whether ERT can be safely applied as additional or alternative therapy following HSPC-LVGT in a murine model of Pompe disease. We found that lentiviral expression at subtherapeutic dose was sufficient to induce tolerance to the transgene product, as well as to subsequently administered ERT. Immune tolerance was established within 4–6 weeks after gene therapy. The mice tolerated ERT doses up to 100 mg/kg, allowing ERT to eliminate glycogen accumulation in cardiac and skeletal muscle and normalizing locomotor function. The presence of HSPC-derived cells expressing GAA in the thymus suggested the establishment of central immune tolerance. These findings demonstrate that lentiviral gene therapy in murine Pompe disease induced robust and long-term immune tolerance to GAA either expressed by a transgene or supplied as ERT.

INTRODUCTION

Pompe disease, also called glycogen storage disease type II (GSDII; Online Mendelian Inheritance in Man [OMIM]: 232300), is an autosomal-recessive lysosomal storage disorder caused by deficiency of the lysosomal enzyme acid alpha-glucosidase (GAA). GAA deficiency leads to glycogen accumulation that eventually results in tissue damage and loss of function.[1,2] The most severe form of the disease is classic infantile Pompe disease. This form is characterized by a complete GAA enzyme deficiency. Patients present with a hypertrophic cardiomyopathy and general skeletal muscle weakness shortly after birth. Without treatment, classic infantile patients die within the first year of life of cardiorespiratory failure.[3,4] Partial GAA deficiency with residual enzymatic activity leads to a milder and less progressive phenotype.[5,6] The age of onset in these patients can vary from early childhood to late adulthood, and symptoms mainly include progressive proximal muscle weakness, while cardiac involvement is rare.[7,8]

Clinical trials for enzyme replacement therapy (ERT) with recombinant human GAA (rhGAA) derived from either rabbit milk[9] or Chinese hamster ovary (CHO) cells[10,11] have shown reversal of cardiomyopathy, improved skeletal muscle function, and enhanced survival in classic infantile patients.[12–14] Improved skeletal muscle strength and function were also observed in late-onset patients after 3 years of treatment.[15] These findings paved the way for approval of ERT for Pompe disease (Myozyme; Genzyme Corporation) in 2006.[16–18]

Despite the benefit of ERT, there are a number of drawbacks related to this treatment. RhGAA is inefficiently delivered to target tissues, for example, skeletal muscle tissue, leading to a heterogeneous response

Received 24 November 2021; accepted 29 April 2022;
https://doi.org/10.1016/j.omtm.2022.04.016.

[8]Present address: Child Neurology, Emma Children's Hospital, Amsterdam University Medical Centers, Vrije Universiteit and Amsterdam Neuroscience, 1081HV Amsterdam, the Netherlands

[9]These authors contributed equally

[10]These authors contributed equally

[11]These authors contributed equally

Correspondence: W.W.M. Pim Pijnappel, PhD, Erasmus University Medical Center, 3015GE Rotterdam, the Netherlands.
E-mail: w.pijnappel@erasmusmc.nl

 © 2022 The Author(s).
This is an open access article under the CC BY license (http://creativecommons.org/licenses/by/4.0/).

in patients.[12,18–29] Moreover, long-term outcome studies in infantile patients whose life expectancy increased because of ERT have shown that glycogen storage in the brain also affects cognitive function. rhGAA is not able to cross the blood-brain barrier, preventing efficient treatment of glycogen accumulation in the CNS and peripheral nervous system.[30–32] Another limitation of ERT is the formation of neutralizing antibodies to rhGAA, which can block its catalytic activity or block the uptake of rhGAA into cells.[33,34] Accumulating evidence indicates that the efficacy of ERT in classic infantile patients can be attenuated when high sustained antibody titers are formed.[35–38] In particular, cross-reactive immunological material (CRIM)-negative patients, in whom no detectable GAA protein is produced, are at risk for developing high sustained antibody titers and have a poor prognosis.[36–38] However, also CRIM-positive classic infantile patients and some childhood- and adult-onset patients can develop high anti-rhGAA antibody titers.[39–41] These observations have recently initiated the development of immunomodulatory protocols aimed at reducing antibody formation in classic infantile patients.[42,43] Nevertheless, variable results with respect to the efficacy of these immunomodulatory protocols have been reported.[44–49]

Hematopoietic stem and progenitor cell (HSPC)-mediated lentiviral gene therapy provides an attractive alternative treatment for lysosomal storage diseases. Clinical trials using this approach have been initiated for several disorders, including Wiskott-Aldrich syndrome,[50] β-thalassemia major,[51] X-linked adrenoleukodystrophy,[52] and metachromatic leukodystrophy.[53] Although previous trials using gamma-retroviral backbones resulted in several cases of insertional mutagenesis-induced leukemia,[54,55]third-generation lentiviral backbones have proved to be safe in clinical trials with more than 7 years follow up.[53]

Previously, our laboratory generated a *Gaa* knockout (KO) ($Gaa^{-/-}$) mouse model that has a complete GAA deficiency, resulting in tissue pathology and symptoms indicative of classic infantile Pompe disease.[56–58] Using this mouse model, we demonstrated that HSPC-mediated lentiviral gene therapy with human GAA resulted in increased GAA enzyme activity in host tissues, reduction of glycogen accumulation, and improved cardiac and motor function.[59,60] Although intravenous treatment with rhGAA resulted in a strong immune response in $Gaa^{-/-}$ mice, characterized by high anti-rhGAA antibody titers and anaphylactic shock,[9,61] we and others previously reported that HSPC-mediated lentiviral gene therapy can prevent anti-GAA antibody formation for up to 5 injections of ERT.[59,62] However, whether this prevention of antibody formation is also robust in the long term, whether it can induce tolerance to higher doses of ERT, and whether it allows additional treatment with ERT to be efficacious is still unknown. This information is highly relevant for future clinical development of gene therapy for Pompe disease, because treatment is essential for classic infantile patients to survive. It is therefore essential to be able to treat patients with ERT in cases in which HSPC-mediated lentiviral gene therapy has insufficient efficacy.

In this study, we investigated the parameters that are relevant for robust immune tolerance to ERT, reversal of disease symptoms and survival of mice. We show that HSPC-mediated lentiviral gene therapy, even when applied at subtherapeutic doses, was sufficient to induce robust immune tolerance to the transgene product and to complementary ERT. Immune tolerance commenced within 4–6 weeks after transplantation, when hematopoietic reconstitution was still incomplete, and it allowed ERT to reduce glycogen accumulation in cardiac and skeletal muscle tissue. This indicates that partial immune reconstitution by gene-modified donor cells was already sufficient for effective immune tolerance induction. Moreover, gene-modified hematopoietic cells and GAA protein were present in the thymus, suggesting that central immune tolerance occurred. Together, this study indicates that HSPC-mediated lentiviral gene therapy induces immune tolerance to GAA and that subsequent treatment with ERT is safe and efficacious, offering a promising scenario for the clinical development of lentiviral gene therapy for the treatment of Pompe disease.

RESULTS

Effect of viral gene dose on immune tolerance induction

In previous studies, we and others pointed to the potential of lentiviral gene therapy to induce immune tolerance to rhGAA.[59,62] However, the relationship between immune tolerance induction and the lentiviral dose remained unclear. To test this, immune tolerance to rhGAA as a result of gene therapy with a multiplicity of infection (MOI) of 2, which is only partially efficient in reducing glycogen accumulation in our Pompe mouse model (see below), was compared with gene therapy at an MOI of 20 (Figures 1A and 1B). Gene therapy at an MOI of 2 resulted in an average vector copy number (VCN) of 3 per genome and an average donor chimerism of 46% (Figures S1A and S1B), while gene therapy at an MOI of 20 with the same preconditioning dose led to an average VCN of 8 per genome and an average donor chimerism of 43% (Figures S1A and S1B).

LV-SF-GAAco-treated mice were treated weekly with ERT at 20 mg/kg for 10 weeks starting 6 weeks after transplantation without problems. In contrast, LV-SF-GFP-treated mice developed an anaphylactic shock and died when more than two weekly rhGAA injections were administered.[9,63] During these two weekly injections, LV-SF-GFP-treated mice developed high sustained antibody titers to rhGAA (Figure 1B, open squares). Gene therapy with the LV-SF-GAAco vector at an MOI of 20, however, prevented anti-rhGAA antibody formation in response to ERT for the entire treatment period of 10 weeks (Figure 1B, open triangles; titers were <1:300, which is within the background levels of the assay, indicated in gray). Interestingly, prevention of anti-rhGAA antibody formation in response to ERT was also achieved at a low viral dose (MOI = 2) (Figure 1B, circles). No anti-rhGAA antibody formation was detected in LV-SF-GAAco-treated mice (MOI = 2) in the absence of ERT. These observations indicate that HSPC-mediated lentiviral gene therapy with human GAA can already induce immune tolerance at a subtherapeutic lentiviral dose. Therefore, lentiviral gene therapy at an MOI of 2 was applied in all subsequent experiments.

Timing of immune tolerance induction

The time needed by lentiviral gene therapy to induce immune tolerance was investigated by comparing the interval between gene

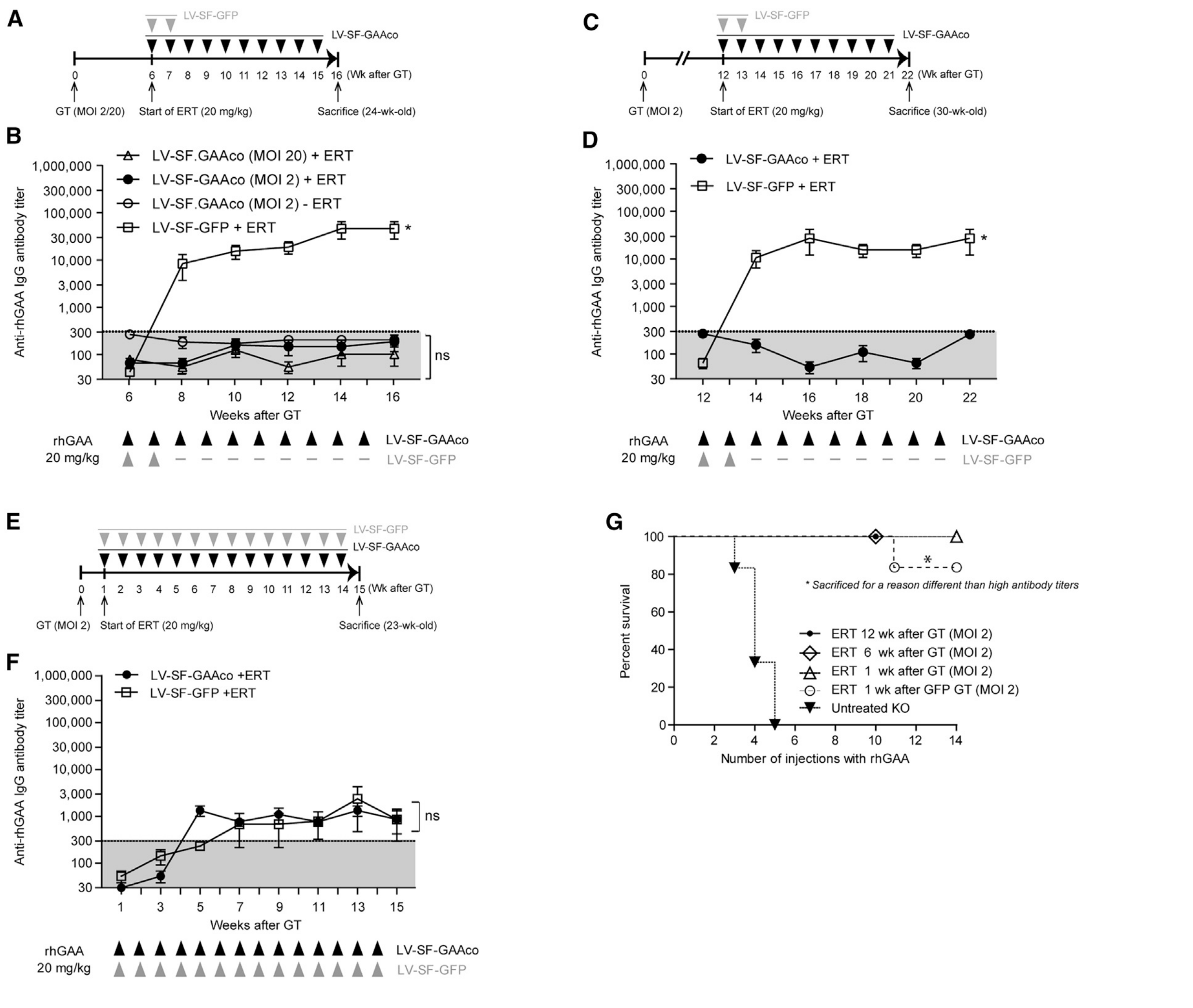

Figure 1. Timing of immune tolerance induction by HSPC-mediated lentiviral gene therapy

(A–F) Lentiviral gene therapy in 8-week-old *Gaa*$^{-/-}$ mice was combined with or without ERT injections (weekly 20 mg/kg, intravenous [i.v.]; indicated by arrowheads) starting 6 weeks (A and B), 12 weeks (C and D), or 1 week (E and F) after gene therapy. In all cases, preconditioning consisted of total-body irradiation at 6 Gy. The number of ERT doses in LV-SF-GFP-treated mice was adjusted to avoid anaphylactic shock and death. (B, D, and F) Anti-rhGAA antibody titers determined by ELISA. Gray zones indicate the technical background of the ELISA assay. Arrowheads below x axis indicate the timing of ERT injections after lentiviral gene therapy. (G) Effect of immune tolerance induction on ERT-induced death in mice receiving lentiviral gene therapy using LV-SF-GAAco or LV-SF-GFP (1 week interval) at an MOI of 2 following the schemes shown in (A), (C), and (E). *Gaa*$^{-/-}$ mice (indicated as KO) served as control and did not receive gene therapy. One mouse of 1 week interval was sacrificed after the 11th injection because of thymic proliferation. Data are represented as mean ± SEM; n = 6 per group, repeated-measures ANOVA with post hoc Tukey's analysis. *$p < 0.05$. ns, not significant; GT, gene therapy; MOI, multiplicity of infection; ERT, enzyme replacement therapy; rhGAA, recombinant human acid α-glucosidase; IgG, immunoglobulin G; Δ + Δ, ERT injections; —, PBS injections.

therapy and the start of ERT of 1, 6, and 12 weeks (Figures 1C and 1E). After an interval of 12 weeks, which ensures full hematopoietic reconstitution after transplantation,[64,65] ERT treatment did not elicit antibody formation in LV-SF-GAAco-treated mice, whereas it caused significant antibody formation in LV-SF-GFP-treated mice (Figure 1D). This was similar to what was observed after a 6 week interval (Figure 1B). When the interval was shortened to 1 week, anti-rhGAA antibodies were formed in LV-SF-GAAco-treated mice (both at an MOI of 2) in response to ERT injections, but at low titers of up to 1:3,000 (Figures 1E and 1F) compared with previous observed titers of 1:30,000 (Figures 1B and 1D), which remained stable until the end of the experiment. Interestingly, low antibody titers in response to ERT administered 1 week after gene therapy were also observed in LV-SF-GFP-treated mice (Figure 1F), suggesting that ERT administration shortly after preconditioning followed by allogeneic hematopoietic stem cell transplantation prevents the induction of anti-rhGAA antibodies to some extent. An expanded ERT scheme with 14 injections was applied in both groups to exclude any delayed immune response, which did not occur. No significant difference in VCN in LV-SF-GAAco-treated mice was observed between any of

the ERT intervals used (Figures S1A, S1C, S1E, and S1G), while chimerism levels of 64% obtained with a 12 week interval were significantly higher than those obtained using a 6 week (39%) or 1 week (36%) interval (Figures S1B, S1D, S1F, and S1H). This difference in chimerism did, however, not seem to affect immune tolerance induction against rhGAA. For LV-SF-GFP, no significant differences in VCN or chimerism where observed between any of the intervals used (Figures S1I and S1J). Together, these results indicate that lentiviral gene therapy starting at least 6 weeks prior to ERT can fully prevent formation of anti-rhGAA antibodies.

Survival in immune-tolerant mice

Antibody formation against rhGAA in *Gaa*$^{-/-}$ mice after more than three injections of ERT is known to result in anaphylactic shock and death of the mice.[9,61,63] In line with this, we observed that the mice started to die after the third ERT injection, while none of the mice survived more than five injections (Figure 1G). In contrast, gene therapy with LV-SF-GAAco prevented anti-rhGAA antibody formation, and resulted in 100% survival of mice that received 10 weekly ERT injections starting 6 or 12 weeks after transplantation. In addition, mice that received LV-SF-GAAco and ERT at an interval of 1 week survived all 14 injections, indicating that anti-rhGAA immune mediated responses were sufficiently reduced to prevent anaphylactic shock-related death, despite the fact that low anti-rhGAA antibody titers were still present.

Effect of conditioning for lentiviral gene therapy on immune responses

To test the effect of the pre-transplantation conditioning regimen on immune tolerance induction, total-body irradiation (TBI) prior to the gene therapy was reduced from 6 to 2 Gy (Figure 2A). When ERT with rhGAA was commenced 4 weeks after transplantation, all LV-SF-GAAco-treated mice died of anaphylactic shock within the first hour after ERT administration (data not shown). When antibody levels were assessed within 2 weeks after LV-SF-GAAco gene therapy without any ERT administration, antibody titers up to 1:100,000 to the transgene product were observed (Figure 2B). After 4 weeks, titers in these mice, without providing additional ERT, plateaued at 1:1,000,000. This immune response to the transgene product was the result of failed engraftment due to insufficient preconditioning, as judged by very low VCNs in bone marrow (Figures 2C and 2D; compare VCN of 0.005 after 2 Gy with VCN of 3 at 6 Gy; Figures S1A and S1B). Anti-rhGAA antibody levels in LV-SF-GFP mice were similar in mice preconditioned with either 6 or 2 Gy (Figures 1D and 2B). This indicates that a preconditioning regimen of 2 Gy TBI is insufficient to allow successful engraftment in our mouse model of Pompe disease, and that sufficient preconditioning is essential for immune tolerance induction.

Immune tolerance against high doses of ERT

Recent studies suggest that a higher dose of ERT may improve outcome in classic infantile patients.[66] To investigate the effect of high doses of ERT on immune tolerance induction by lentiviral gene therapy, mice were treated with a subtherapeutic lentiviral

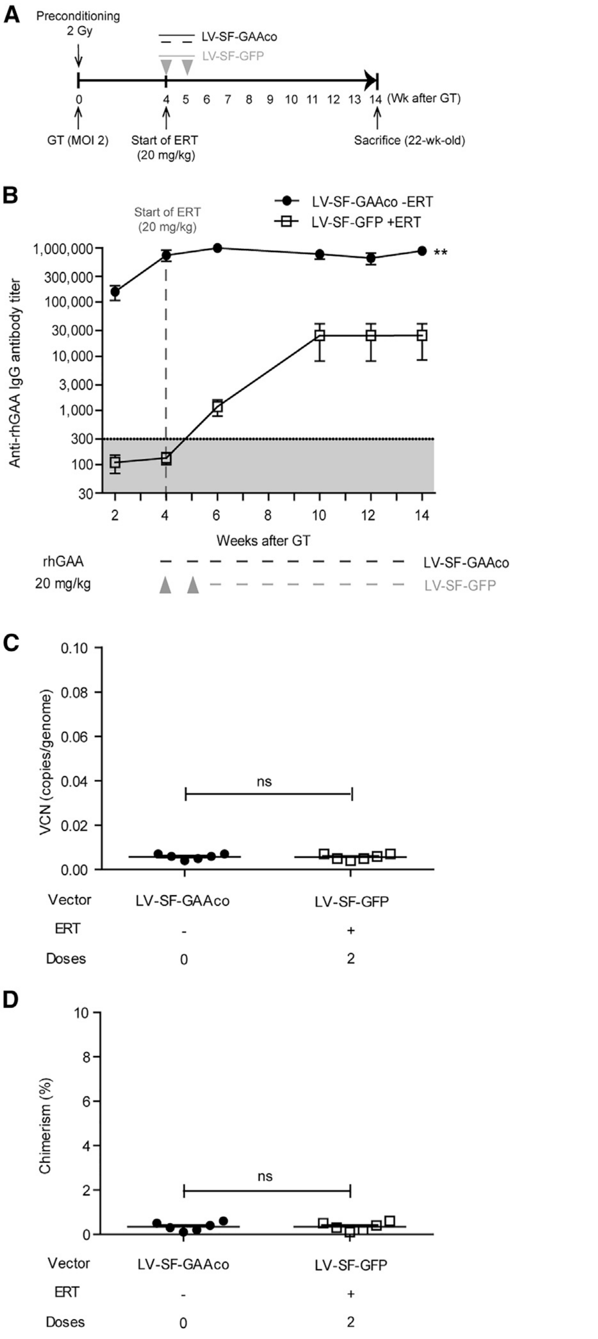

Figure 2. Sufficient preconditioning is required for successful engraftment

(A) Experimental design. Eight-week-old *Gaa*$^{-/-}$ mice received weekly ERT injections starting 4 weeks after lentiviral gene therapy using preconditioning with total-body irradiation at 2 Gy. (B) Anti-rhGAA IgG titers determined by ELISA. Bone marrow was collected at the end of the experiment, and vector copy number (C) and chimerism (D) were determined by *HIV* and *Sry* qPCR, respectively, and normalized using *Gapdh*. Data are represented as mean ± SEM. n = 6 per group. **$p < 0.01$. ns, not significant.

dose (MOI = 2) of LV-SF-GAAco followed by weekly injections of 100 mg/kg rhGAA instead of 20 mg/kg, starting 4 weeks after gene therapy (Figure 3A). Complete prevention of antibody formation to both the transgene product and a high dose of ERT was observed in the LV-SF-GAAco-treated mice, while the same ERT regimen applied to mice treated with LV-SF-GFP elicited antibody formation against rhGAA (Figure 3B). The VCN in LV-SF-GAAco-treated mice with ERT injections of 100 mg/kg was about 4 with 49% chimerism (Figures S2A and S2B), similar to the values obtained in LV-SF-GAAco mice treated with ERT injections of 20 mg/kg. These results show that lentiviral gene therapy induces immune tolerance to ERT with rhGAA irrespective of the ERT dose. Weekly doses as high as 100 mg/kg rhGAA were tolerated.

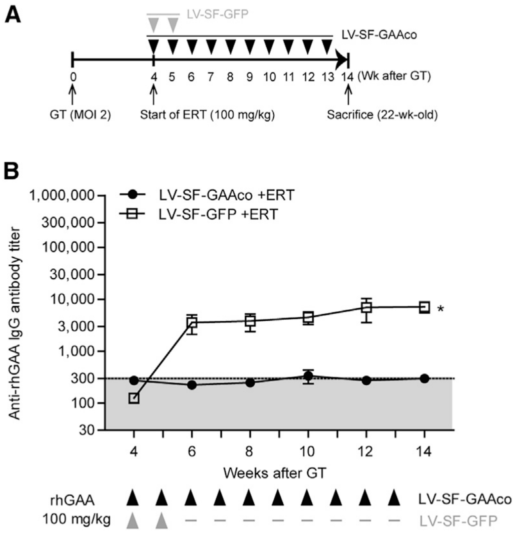

Figure 3. Induced immune tolerance is still functional after increased ERT dosing

(A) Experimental design. Eight-week-old *Gaa*$^{-/-}$ mice received weekly ERT injections of 100 mg/kg rhGAA, starting 4 weeks after lentiviral gene therapy. Preconditioning consisted of total body irradiation at 6 Gy. (B) Anti-rhGAA antibody titers determined by ELISA. *$p < 0.05$. Data are represented as mean ± SEM.

Therapeutic efficacy of ERT after immune tolerance induction

As it cannot be excluded that in the future patients with an insufficient effect of gene therapy might need additional ERT, we tested whether ERT remains efficacious after treatment with gene therapy. Glycogen levels, enzyme activity, and rotarod performance were assessed in mice 3.5–4 months after treatment with gene therapy (at the age of 22–24 weeks) and with subsequent ERT treatment dosed at 20 or 100 mg/kg. Data are derived from the same experiments and mice as described in Figures 1A, 1B, 3A, and 3B, with both intervals leading to a full prevention of antibody formation (for treatment schedule, see Figures 1A and 3A, respectively). Subtherapeutic LV-SF-GAAco treatment (using an MOI of 2) alone resulted in an increased GAA enzymatic activity in the heart, quadriceps femoris, and diaphragm (Figures 4A and 4B) but only a partial reduction of glycogen levels (Figures 4C and 4D). In addition, we observed a partial restoration of motor performance tested on a rotarod (Figures 4E and 4F). Treatment with ERT after gene therapy provided additional efficacy. At a dose of 20 mg/kg, efficacy was improved, but glycogen levels were still not completely reduced to the levels in wild-type (WT) mice (Figures 4C and 4E). Increasing the dose to 100 mg/kg resulted in full correction of glycogen levels and rotarod performance (Figures 4D and 4F). Taken together, this demonstrates that immune tolerance induction by lentiviral gene therapy allowed ERT to be introduced and to be effective in normalizing glycogen levels in heart and skeletal muscles and in restoring skeletal muscle function.

Central immune tolerance via the expression of GAA in the thymus

Central immune tolerance to endogenous proteins is established in the thymus via negative selection of T cells that are able to interact with endogenous antigens to distinguish self from foreign antigens. Endogenous expression of proteins in the thymus is therefore a prerequisite for the establishment of immune tolerance for these proteins. To test whether HSPC-mediated lentiviral gene therapy could induce central immune tolerance, we tested whether the GAA transgene was expressed in the thymus. To this end, we assessed GAA protein expression and enzyme activity in the thymus of LV-SF-GAAco or LV-SF-GFP-treated mice, that did or did not receive additional ERT treatment (Figure 5A). Western blot analysis (Figures 5B and 5C) and enzyme activity assays (Figure 5D) indicated the presence of human GAA in the thymus of mice treated with LV-SF-GAAco. No significant differences in either GAA protein level or GAA enzyme activity could be observed in mice treated with or without 10 additional ERT injections starting 6 weeks after gene therapy with LV-SF-GAAco (Figures 5C and 5D). Reducing the interval between gene therapy and ERT to 1 week, which was not sufficient to establish a complete immune tolerance, resulted in a 4-fold reduction of GAA levels and activity. No human GAA protein was detected in the thymus of LV-SF-GFP-treated mice after 2 injections of ERT, and also not in untreated KO mice or WT mice (Figures 5C and 5D). VCNs in the thymus and bone marrow (Figures 5E and 5F; both VCNs of 6 copies per genome) indicated successful engraftment of GAA-corrected cells into both tissues, suggesting that the observed GAA expression in the thymus resulted from the migration of GAA-corrected HSPCs from the bone marrow into the thymus. Together, these data suggest that the immune tolerance obtained via lentiviral gene therapy is a result of transgene expression in the thymus, inducing central tolerance against GAA.

DISCUSSION

Enzyme replacement therapy has improved outcomes among patients with lysosomal storage diseases, but antibodies to the infused recombinant protein that either neutralize its enzyme activity or inhibit tissue uptake, particularly in classic infantile CRIM-negative patients, can interfere with the clinical outcome. This has also been observed in mouse models of Pompe disease.[67] Hence, immunomodulatory agents and gene therapy protocols have been investigated to improve outcomes.[68,69] Allogeneic hematopoietic stem cell transplantation has

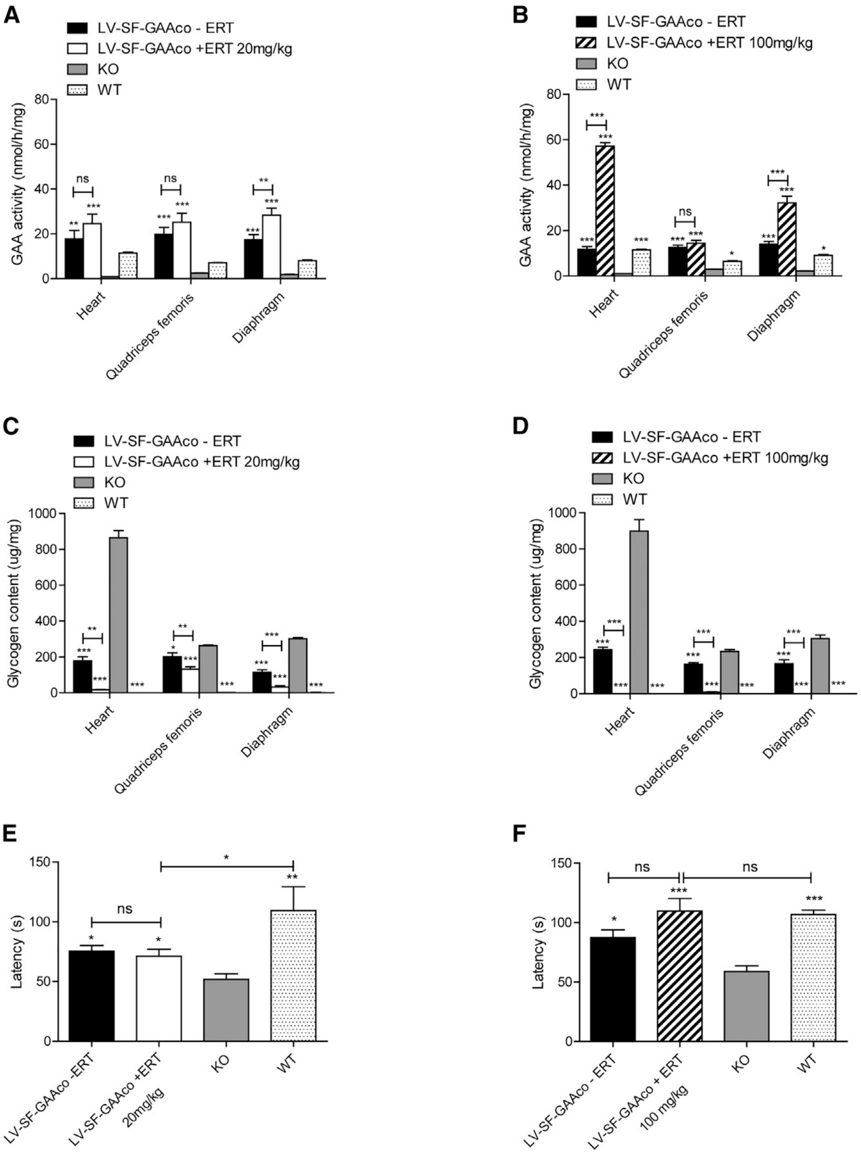

Figure 4. Efficacy of ERT after lentiviral gene therapy

GAA activity, glycogen clearance, and rotarod performance in mice treated with LV-SF-GAAco gene therapy and weekly i.v. injections of 20 mg/kg starting 6 weeks after gene therapy (A, C, and E) or 100 mg/kg rhGAA starting 4 weeks after gene therapy (B, D, and F). Data are derived from the same experiments as described in Figures 1A, 1B, 3A, and 3B, with both intervals leading to a full prevention of antibody formation. LV-SF-GAAco-treated mice that received weekly PBS injections instead of ERT were included to evaluate the effect of gene therapy alone (black columns). Age-matched untreated $Gaa^{-/-}$ and wild-type mice served as controls. GAA enzymatic activity (A and B), glycogen levels (C and D), and motor function as measured by latency on a rotarod (E and F) were determined after the 10th injection of ERT. Data are represented as mean ± SEM; n = 6 per group in (A), (C), and (E); n = 5 per group in (B) and (D); n = 8 per group in (F). Asterisks without bars indicate comparison with KO mice; additional comparisons are indicated with bars. *p < 0.05, **p < 0.01, and ***p < 0.001. ns, not significant.

been shown to be an effective therapy for rapid immune tolerance induction in inborn metabolic diseases, such as Hurler disease.[70] In this study, we investigated the properties and mechanism of immune tolerance induction by lentiviral gene therapy in a mouse model of Pompe disease. HSPC-mediated lentiviral gene therapy, even at a low subtherapeutic dose (MOI = 2), was able to prevent antibody formation to the GAA transgene product and to additional treatment with rhGAA. Partial chimerism of gene-modified donor cells was sufficient for sustained immune tolerance induction and could be achieved during hematopoietic reconstitution. Subtherapeutic lentiviral gene therapy prevented ERT-induced anaphylactic death in mice. It also allowed long-term treatment with high-dose ERT without the induction of an immune response, resulting in complete glycogen clearance and normalization of muscle function. We showed that gene-modified cells were present in the thymus, where they contributed to GAA protein production, which may have played a pivotal role in central immune tolerance induction to GAA. These results emphasize that HSPC-mediated lentiviral gene therapy may not only serve as an alternative treatment option for Pompe disease, but also as an immunomodulatory approach to overcome the immunological complications associated with ERT. This would be especially important for classic infantile patients because of the risk for high anti-GAA antibody titers after ERT treatment, which is less prominent in the majority of late-onset patients.[39,40,71]

In the clinical setting where classic infantile patients may be treated with gene therapy in the future, some patients might benefit from ERT before, during, or after HSPC-mediated lentiviral gene therapy, as has been investigated for Hurler syndrome patients.[70,72] In case gene therapy might not provide the desired effect, additional ERT infusions may be required. As classic infantile Pompe patients rapidly deteriorate in the absence of effective treatment, it will be essential to establish the shortest possible time frame between lentiviral gene therapy and the potential initiation of ERT, while ensuring a robust immune tolerance induction at the same time. In our effort to determine the minimum time required to induce efficient immune tolerance after gene therapy, we showed that during hematopoietic reconstitution, which normally takes 8–12 weeks in mice,[64,65] anti-rhGAA antibody formation could be completely prevented when ERT challenges were started 4–6 weeks after lentiviral gene therapy. Shortening the interval to just 1 week did not prevent antibody formation completely, but we found

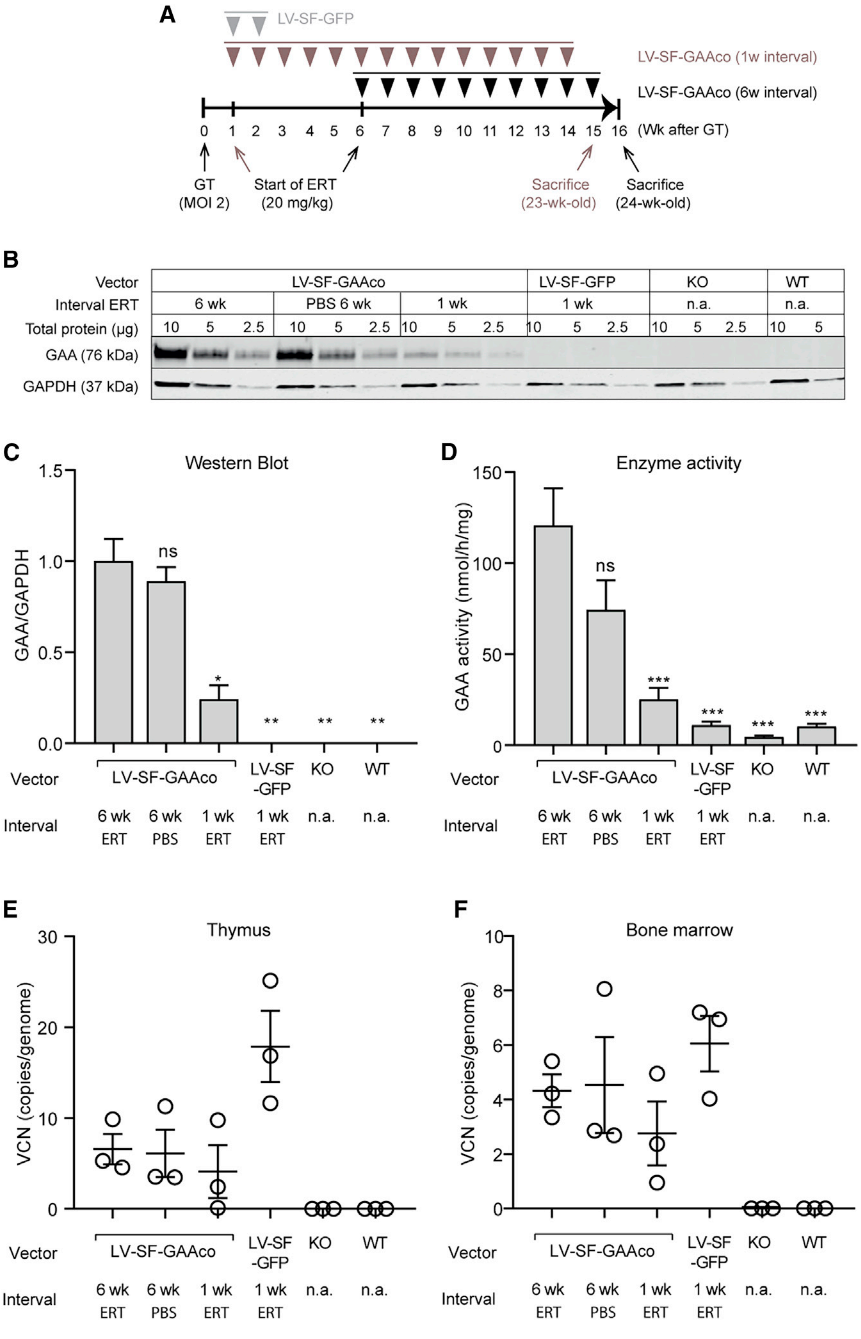

Figure 5. Protein expression and enzyme activity in thymus

Thymi of mice were assessed for transgene expression to assess the potential involvement of central immune tolerance induction. Groups include mice receiving LV-SF-GAAco with or without subsequent ERT injections or receiving LV-SF-GFP with two subsequent ERT injections. LV-SF-GAAco-treated mice that received weekly PBS injections were included to evaluate the effect of gene therapy alone. Age-matched untreated $Gaa^{-/-}$ and wild-type mice served as controls. (A) Schematic representation of the experimental setup of mice receiving gene therapy in combination with ERT at the indicated intervals. (B) Western blot analysis of human GAA and GAPDH at different total protein amounts (micrograms) as indicated (2.5 μg not shown in WT mice). (C) Western blot quantification of human GAA relative to GAPDH expression, normalized to the GAA expression of LV-SF-GAAco in combination with ERT at a 6 week interval. (D) GAA enzyme activity measured using the 4-methylumbelliferone (4-MU) assay, normalized for total protein levels. (E and F) Vector copy number (VCN) in thymus (E) and bone marrow (F) as described above. Data are represented as mean ± SEM; n = 3 per group; tissues of the same mice were used for VCN, western blot, and enzyme activity analysis. Asterisks indicate comparison with LV-SF-GAAco-treated mice with subsequent ERT injections after a 4 week interval. *$p < 0.05$, **$p < 0.01$, and ***$p < 0.001$. ns, not significant.

lower antibody titers compared with mice treated with ERT without gene therapy. In addition, these lower antibody titers did not result in anaphylactic shock and the death of mice, in contrast to mice that were treated with ERT without gene therapy. Lower antibody titers to rhGAA were also observed when GFP-transduced mice were challenged with ERT starting 1 week after gene therapy. This might indicate that exposure to a foreign antigen during early hematopoietic reconstitution is likely to prevent antibody formation against ERT and the transgene product to some extent, which is in agreement with previous studies showing antigen-induced tolerance.[73,74] In contrast to previous studies that used an interval of 12 weeks after HSPC transplantation to allow full hematopoietic reconstitution, here we show that immune tolerance can even be induced during hematopoietic immune reconstitution.[62,75] As the disease symptoms in classic infantile Pompe patients rapidly progress, patients should be treated as soon as possible after diagnosis, and starting ERT injections prior to gene therapy might be preferable. However, it is important to investigate whether in that setup, no pre-existing CD8 positive effector T cells (CTLs) would be present which could hamper the efficiency of lentiviral gene therapy, as has been previously shown for mucopolysaccharidosis I.[76] Future experiments should therefore focus on the conditions required to combine ERT with gene therapy when ERT is provided prior to gene therapy.

To assess the effect of full or partial preconditioning on engraftment and immune tolerance induction, we tested the effect of a less intensive TBI preconditioning protocol. Reduction from 6 to 2 Gy resulted in no engraftment and a rapid development of high antibody titers to the transgene product, which might be explained by insufficient

immune suppression or insufficient myeloablation, resulting in a lack of space in the bone marrow to allow engraftment. This suggests that the preconditioning regimen is pivotal to ensure full engraftment and to induce immune tolerance. Future experiments are required to assess critical preconditioning requirements with more clinically relevant preconditioning regimens like busulfan- or treosulfan-based regimens with or without additional immunosuppression. These are immunosuppressive and myeloablative regimens that have a similar ablative nature compared with irradiation.[77] Extensive experience in the clinic over the years as well as therapeutic drug monitoring resulted in improved outcome of these regimens and are at this moment the most commonly used agents in the clinic for current gene therapy trials in patients with inborn errors.[75,78–80]

Recent reports suggest that the recommended ERT dose currently used for classic infantile patients (20 mg/kg biweekly) is insufficient to correct disease symptoms and that increasing the dose to weekly infusions of 40 mg/kg provides a better prognosis.[66,81,82] Weekly injections of 20 mg/kg in our $Gaa^{-/-}$ mouse model supported these findings and showed that 20 mg/kg did not fully reverse the phenotype, in line with previous studies.[61] Therefore, the question was raised whether the increased doses of ERT, and thereby increased antigen exposure, would interfere with immune tolerance induced by gene therapy. We showed that subtherapeutic lentiviral gene therapy prevented antibody responses and death even when ERT was dosed at 100 mg/kg weekly. In addition, this dose was effective in normalizing glycogen levels and muscle performance on a rotarod. Even though we show that higher doses of ERT are tolerated after gene therapy and thereby show that the tolerance is not limited to administration of low concentrations of the recombinant enzyme, reducing the frequency or dose of ERT would also be clinically relevant. Therefore, future studies should determine whether lentiviral gene therapy, in case it turns out to be less than efficient, would have the additional benefit of allowing ERT treatment with reduced frequency or dose, without compromising its efficiency on glycogen clearance.

Central tolerance is induced by elimination of auto-reactive CD8+ T cells in the thymus that respond to self-antigens with high affinity.[83–86] In line with previous studies,[85,87] our data suggest that the mechanism by which lentiviral gene therapy results in immune tolerance is via the induction of central tolerance. Lentiviral gene therapy results in expression of the GAA transgene by HSPC-derived cells, which could also reside in the thymus. The recognition of the transgene product by the developing T cells as an endogenous protein would result in negative selection of the T cells and the induction of immune tolerance. Even though we do not show direct evidence of which cells are expressing GAA in the thymi, the lack of GAA detection in thymi from mice treated with GFP gene therapy in combination with ERT indicates that the GAA observed is originating from HSPC-derived cells. It is tempting to speculate that the hGAA is expressed by thymic epithelial cells, eliminating GAA-reactive T cells, which should be confirmed in future studies. Immune tolerance can also be induced by applying liver-specific expression through adeno-associated virus (AAV) vectors, which may also enhance the therapeutic efficacy of ERT.[63,67,88–90] This type of gene therapy, however, acts in a different way and involves peripheral immune tolerance, a process mediated by regulatory T cells. It remains to be determined whether T regulatory cells also play a role in lentiviral-mediated gene therapy. The establishment of central immune tolerance via HSPC-mediated lentiviral gene therapy could provide long-term benefit for patients with Pompe disease who need ERT with rhGAA at some point in time in addition to gene therapy.

The importance of managing immune reactions to ERT in patients with classic infantile Pompe disease is highlighted by the variety of current strategies for immunomodulation involving treatment with rituximab, methotrexate, bortezomib, cyclophosphamide, mycophenolate, or sirolimus.[44,47–49,91,92] This is especially important in classic infantile CRIM-negative patients, who do not express GAA protein and generally develop high antibody titers that may neutralize ERT. Current immunomodulation strategies have variable success in inducing immune tolerance to ERT. We showed here that an additional advantage of HSPC-mediated lentiviral gene therapy for Pompe disease is that it induces immune tolerance to rhGAA protein. This not only allows additional treatment with ERT in patients in whom gene therapy would have insufficient efficacy, but it also prevents antibody formation, likely allowing a better clinical response to treatment with either lentiviral gene therapy or ERT.

MATERIALS AND METHODS

Animals and procedures

All animal procedures in this study conformed to Dutch law for the protection and use of animals for scientific procedures and were approved by the Animal Experiments Committee (DEC) in the Netherlands. Immunocompetent $Gaa^{-/-}$ knockout mice in an FVB/N background were used for all experiments.[56] $Gaa^{-/-}$ mice are completely deficient of GAA enzymatic activity and were generated as previously described.[56] Age-matched FVB/N mice were purchased from Charles River (Wilmington, MA) as wild-type controls. All mice were housed under specific pathogen-free (SPF) conditions in the Animal Experimental Center at the Erasmus MC (EDC) according to standard procedures, which included a 12 h light-dark cycle and *ad libitum* diet. Mice were deprived of food 15 h pre-sacrifice to deplete cytoplasmic glycogen.[9] Subsequently, mice were anesthetized with ketamine (10%; Alfasan, Woerden, the Netherlands) and Sedator (1 mg/mL; Eurovet, Bladel, the Netherlands) and sacrificed by intracardiac perfusion with 50 mL PBS to remove blood. Relevant tissues, which included heart, diaphragm, quadriceps femoris, bone marrow, and thymus, were harvested, snap-frozen in liquid nitrogen, and stored at −80°C until further analysis.

Lentiviral vector construction and production

Codon-optimized human *GAA* (*GAAco*; GenScript, Piscataway, NJ) was cloned into the previously described[59] third-generation self-inactivating (SIN)[93] lentiviral vector pRRL.PPT.SF.GFP.bPRE4*.SIN (LV-SF-GFP) by replacing GFP using AgeI and SbfI restriction sites, resulting in the lentiviral vector LV-SF-GAAco. Expression of the

transgene was driven by the spleen focus-forming virus (SFFV) promoter. Lentiviruses were produced in HEK293T cells by calcium phosphate cotransfection with plasmids pMDL-g/pRRE, pMD2-VSVg, and pRSV-Rev and were concentrated by ultracentrifugation (Beckman, SW32TI rotor) at 20,000 rpm for 2 h at 4°C.[94,95] Lentiviral titers were determined in transduced HeLa cells, by measuring vector copy numbers 5 days post-transduction using quantitative polymerase chain reaction (qPCR) with primers targeting the U3 and Psi sequences of *HIV* (listed in Table S1). A standard curve was prepared using HeLa cells carrying on average 1 copy of integrated lentiviral vector per genome. Final titers were determined as the average VCNs multiplied by the cell number (2×10^5) and fold dilution. Titers of approximately 10^8 transduction units/mL were routinely obtained for all viral vectors.

Lentiviral hematopoietic stem cell transduction and transplantation procedures

Bone marrow cells were extracted from the tibiae and femora of 8-week-old male $Gaa^{-/-}$ donor mice and enriched through lineage depletion (Lin^-) using the Mouse Hematopoietic Progenitor Cell Enrichment Set (BD Sciences, San Jose, CA). After enrichment, Lin^- cells were seeded in 6-well plates at a density of 10^6 cells/mL in StemMACS HSPC expansion media (Miltenyi Biotec, Leiden, the Netherlands), supplemented with the following growth factors: murine thrombopoietin (100 ng/mL), murine stem cell factor (100 ng/mL), and human FMS-like tyrosine kinase 3 murine ligand (50 ng/mL).[59] Cells were transduced overnight with concentrated LV-SF-GAAco at various multiplicities of infection (as specified in the figures) or with LV-SF-GFP at an MOI of 2 as control, and incubated at 37°C with 10% CO_2. The following day, 5×10^5 transduced Lin^- cells were transplanted intravenously through the tail vein into 8-week-old female $Gaa^{-/-}$ recipients, previously subjected to 6 Gy (or 2 Gy as specified in one experiment) sublethal total-body irradiation using the Gammacell 40 irradiator (Atomic Energy of Canada).

Treatment of $Gaa^{-/-}$ mice with recombinant human GAA

Depending on the experimental design, enzyme replacement therapy was applied following lentiviral gene therapy. LV-SF-GAAco- and LV-SF-GFP-treated mice were divided into two groups: one group received ERT (marked as "+ ERT" in figures) with recombinant human acid α-glucosidase (rhGAA; Myozyme) intravenously at doses of either 20 or 100 mg/kg, while the other group was subjected to PBS alone (marked as "-ERT" in figures). Plasma was collected at baseline and every other week during the course of ERT to monitor antibody titers. Mice were sacrificed 1 week after the last injection to evaluate the therapeutic effect.

Rotarod

Motor function was evaluated on a rotarod (Panlab, Harvard Apparatus, Holliston, MA), accelerating from 4 to 40 rpm in 5 min. Prior to the experiment, mice were kept in the testing room for at least 15 min to adjust to the environment. A practice session of 3 trial runs was conducted for the mice to adjust to the apparatus, followed by 3 definitive runs with 5 min intervals. The latency to falling off the rotarod was recorded and averaged for each mouse.

Enzyme-linked immunosorbent assay

Enzyme-linked immunosorbent assays (ELISAs) of plasma were performed as described[36,39] with minor adjustments. Blood samples were harvested in dipotassium ethylene diamine tetra acetic acid (EDTA)-coated tubes (K2E tubes, BD microtainer, #365975; Becton and Dickinson) and plasma was collected after centrifugation at 2,000 rpm for 10 min. Samples were diluted 30- to 1,000,000-fold in 3-fold dilution series and analyzed in duplicate. Plates were coated for 2 h using Myozyme (5 μg/mL) at room temperature (RT) and blocked using 1% BSA in PBS overnight at 4°C. After washing, samples diluted 30- to 1,000,000-fold in 3-fold dilution series were incubated in duplicate for 1 h at RT. HRP-goat anti-mouse IgG (1:20,000; #62-6520; Invitrogen) antibodies were incubated for 1 h at RT. After washing, the TMB-2 component microwell peroxidase substrate kit (#50-76-00; KPL) was incubated for 10 min, after which the reaction was stopped using 1 M orthophosphoric acid, and antibody titers were assessed using spectrophotometry at an absorbance of 450 nm. Control plasma samples were incubated on uncoated plates to determine background levels, which ranged between 1:30 and 1:300.[39] In each experiment, rabbit anti-GAA serum (positive control) and plasma from age-matched untreated FVB/N mice (negative control) were included. Titers were defined as the highest dilution at which absorbance was at least twice as high as the averaged value of the negative control plus 10%.

GAA enzymatic activity and glycogen content assays

Tissue aliquots were homogenized completely in 300 μL Milli-Q water (Merck, Millipore) supplemented with protease inhibitors (Complete Protease Inhibitor Cocktail; Roche) using 5 mm stainless steel beads (Qiagen) in the TissueLyser II (Qiagen, Venlo, the Netherlands) for 5 min at 30 Hz. Debris was pelleted by centrifugation at 10,000 rpm for 5 min, and the supernatant was used for GAA enzymatic activity and glycogen content measurements. GAA enzymatic activity was determined in a fluorometric assay using 4-methylumbelliferyl-α-D-glucoside (Sigma-Aldrich, St. Luis, MO) as substrate, as previously described.[96] Glycogen content was quantified by the amount of glucose released from glycogen after conversion by amyloglucosidase and amylase (Roche Diagnostics, Basel, Switzerland) as previously detailed.[9] Both GAA enzymatic activity and glycogen content were normalized to total protein levels determined with the Pierce BCA Protein Assay Kit (Thermo Fisher Scientific, Waltham, MA).

Western blot

Tissue lysates from thymi were obtained as described above. Immunoblotting against GAA was performed as previously described.[97] In short, 10 μg denatured protein with Laemmli sample buffer (62.5 mM Tris-HCL [pH 6.8], 2% SDS, 25% glycerol, 0.01% bromophenol blue, 5% β-mercaptoethanol) was separated on a 4%–15% polyacrylamide gel (Criterion TGX; Bio-Rad) and transferred to a nitrocellulose blotting membrane (GE Healthcare). Membranes

were probed with rabbit anti-GAA (1:1,000; #137068; Abcam) and mouse anti-GAPDH (1:1,000; MAB374; Millipore). Proteins of interest were detected with IRDye 800CW and IRDye 680RD secondary antibodies (LI-COR Biosciences, Lincoln, NE) and were imaged using the Odyssey Infrared Imaging System (LI-COR Biosciences). GAA protein content was quantified using Fiji and normalized against GAPDH.

Quantitative polymerase chain reaction of LV integrations

Genomic DNA was extracted from bone marrow with the NucleoSpin Tissue kit (Macherey-Nagel, Düren, Germany), and used at 100 ng per quantitative polymerase chain reaction. Reactions were performed using iTaq Universal SYBR Green Supermix (Bio-Rad, Hercules, CA). A standard curve for VCNs was prepared using serial dilutions of genomic DNA from transduced mouse 3T3 cells carrying 1 copy of integrated lentiviral vector per genome. Chimerism was determined using primers specific for the *Sry* locus on the mouse *Y* chromosome. Both VCN and chimerism were normalized by mouse *Gapdh*. Bone marrow DNA from untreated male $Gaa^{-/-}$ donor mice was used to establish a reference standard in *Sry* and *Gapdh* qPCRs. Male thymic DNA from an untreated male $Gaa^{-/-}$ donor mouse was used in serial dilutions as a reference for the determination of chimerism in thymus. Reactions were carried out on a CFX96 real-time PCR detection system (Bio-Rad). Primer sequences are shown in Table S1.

Statistics

Statistical analysis was performed with SPSS version 22 (IBM). Repeated-measures ANOVA was applied to detect differences of antibody titer between treatment over the course of ERT, with Tukey's comparison test for individual comparisons among groups. The Mann-Whitney U test was used for comparison of two groups. A p value $< .05$ was considered statistically significant.

SUPPLEMENTAL INFORMATION

Supplemental information can be found online at https://doi.org/10.1016/j.omtm.2022.04.016.

ACKNOWLEDGMENTS

G. Wagemaker was involved in the concept of the study. This work was supported by the China Scholarship Council (Q.L.; File No. 201206240040), the Netherlands Organization for Health Research ZonMw (project number 40-40300-98-07010), the Sophia Foundation (grant S18-59), Metakids (grant 2018-083), the Prinses Beatrix Spierfonds (grant W.OP20-04), and the "Finding a Cure for Hunter Syndrome" Foundation.

AUTHOR CONTRIBUTIONS

N.P.v.T. and W.W.M.P.P. conceived the project. Q.L., E.C.V., F.C., N.P.v.T., and W.W.M.P.P. designed the experiments. Q.L., E.C.V., F.C., and J.M.P. performed experiments. All authors analyzed and interpreted the data. Q.L.,G. Wagemaker, N.P.v.T., A.T.v.d.P., and W.W.M.P.P. obtained funding. Q.L., E.C.V., F.C., N.P.v.T., and W.W.M.P.P. wrote the manuscript. All authors read and approved the final manuscript.

DECLARATION OF INTERESTS

A.T.v.d.P. has received consulting fees from Sanofi Genzyme and has provided consulting services, participated in advisory board meetings, and received grants for premarketing studies and research from industries via agreements between Erasmus MC and the industry. N.P.v.T. is an employee of AVROBIO (Cambridge, MA).

REFERENCES

1. Hirschhorn, R., and Reuser, A.J.J. (2001). Glycogen storage disease type II: acid α-glucosidase (acid maltase) deficiency. In The Metabolic and Molecular Basis for Inherited Disease, Eighth Edition, C. Scriver, A.L. Beaudet, W.S. Sly, and D. Valle, eds. (McGraw-Hill), pp. 3389–3420.
2. van der Ploeg, A.T., and Reuser, A.J. (2008). Pompe's disease. Lancet *372*, 1342–1353.
3. van den Hout, H.M., Hop, W., van Diggelen, O.P., Smeitink, J.A., Smit, G.P., Poll-The, B.T., Bakker, H.D., Loonen, M.C., de Klerk, J.B., Reuser, A.J., et al. (2003). The natural course of infantile Pompe's disease: 20 original cases compared with 133 cases from the literature. Pediatrics *112*, 332–340.
4. Kishnani, P.S., Hwu, W.L., Mandel, H., Nicolino, M., Yong, F., and Corzo, D.; Infantile-Onset Pompe Disease Natural History Study, G (2006). A retrospective, multinational, multicenter study on the natural history of infantile-onset Pompe disease. J. Pediatr. *148*, 671–676.
5. Wokke, J.H., Ausems, M.G., van den Boogaard, M.J., Ippel, E.F., van Diggelene, O., Kroos, M.A., Boer, M., Jennekens, F.G., Reuser, A.J., and Ploos van Amstel, H.K. (1995). Genotype-phenotype correlation in adult-onset acid maltase deficiency. Ann. Neurol. *38*, 450–454.
6. Kroos, M., Hoogeveen-Westerveld, M., van der Ploeg, A., and Reuser, A.J. (2012). The genotype-phenotype correlation in Pompe disease. Am. J. Med. Genet. C Semin. Med. Genet. *160C*, 59–68.
7. Hagemans, M.L., Winkel, L.P., Van Doorn, P.A., Hop, W.J., Loonen, M.C., Reuser, A.J., and Van der Ploeg, A.T. (2005). Clinical manifestation and natural course of late-onset Pompe's disease in 54 Dutch patients. Brain *128*, 671–677.
8. Winkel, L.P., Hagemans, M.L., van Doorn, P.A., Loonen, M.C., Hop, W.J., Reuser, A.J., and van der Ploeg, A.T. (2005). The natural course of non-classic Pompe's disease; a review of 225 published cases. J. Neurol. *252*, 875–884.
9. Bijvoet, A.G., Van Hirtum, H., Kroos, M.A., Van de Kamp, E.H., Schoneveld, O., Visser, P., Brakenhoff, J.P., Weggeman, M., van Corven, E.J., et al. (1999). Human acid alpha-glucosidase from rabbit milk has therapeutic effect in mice with glycogen storage disease type II. Hum. Mol. Genet. *8*, 2145–2153.
10. Fuller, M., Van der Ploeg, A., Reuser, A.J., Anson, D.S., and Hopwood, J.J. (1995). Isolation and characterisation of a recombinant, precursor form of lysosomal acid alpha-glucosidase. Eur. J. Biochem. *234*, 903–909.
11. Van Hove, J.L., Yang, H.W., Wu, J.Y., Brady, R.O., and Chen, Y.T. (1996). High-level production of recombinant human lysosomal acid alpha-glucosidase in Chinese hamster ovary cells which targets to heart muscle and corrects glycogen accumulation in fibroblasts from patients with Pompe disease. Proc. Natl. Acad. Sci. U S A *93*, 65–70.
12. Van den Hout, J.M., Kamphoven, J.H., Winkel, L.P., Arts, W.F., De Klerk, J.B., Loonen, M.C., Vulto, A.G., Cromme-Dijkhuis, A., Weisglas-Kuperus, N., Hop, W., et al. (2004). Long-term intravenous treatment of Pompe disease with recombinant human alpha-glucosidase from milk. Pediatrics *113*, 448–457.
13. Klinge, L., Straub, V., Neudorf, U., Schaper, J., Bosbach, T., Gorlinger, K., Wallot, M., Richards, S., and Voit, T. (2005). Safety and efficacy of recombinant acid alpha-glucosidase (rhGAA) in patients with classical infantile Pompe disease: results of a phase II clinical trial. Neuromuscul. Disord. *15*, 24–31.
14. Kishnani, P.S., Nicolino, M., Voit, T., Rogers, R.C., Tsai, A.C., Waterson, J., Herman, G.E., Amalfitano, A., Thurberg, B.L., Richards, S., et al. (2006). Chinese hamster ovary cell-derived recombinant human acid alpha-glucosidase in infantile-onset Pompe disease. J. Pediatr. *149*, 89–97.

15. Winkel, L.P., Van den Hout, J.M., Kamphoven, J.H., Disseldorp, J.A., Remmerswaal, M., Arts, W.F., Loonen, M.C., Vulto, A.G., Van Doorn, P.A., De Jong, G., et al. (2004). Enzyme replacement therapy in late-onset Pompe's disease: a three-year follow-up. Ann. Neurol. *55*, 495–502.

16. Nicolino, M., Byrne, B., Wraith, J.E., Leslie, N., Mandel, H., Freyer, D.R., Arnold, G.L., Pivnick, E.K., Ottinger, C.J., Robinson, P.H., et al. (2009). Clinical outcomes after long-term treatment with alglucosidase alfa in infants and children with advanced Pompe disease. Genet. Med. *11*, 210–219.

17. van Capelle, C.I., van der Beek, N.A., Hagemans, M.L., Arts, W.F., Hop, W.C., Lee, P., Jaeken, J., Frohn-Mulder, I.M., Merkus, P.J., Corzo, D., et al. (2010). Effect of enzyme therapy in juvenile patients with Pompe disease: a three-year open-label study. Neuromuscul. Disord. *20*, 775–782.

18. Gungor, D., Kruijshaar, M.E., Plug, I., D'Agostino, R.B., Hagemans, M.L., van Doorn, P.A., Reuser, A.J., and van der Ploeg, A.T. (2013). Impact of enzyme replacement therapy on survival in adults with Pompe disease: results from a prospective international observational study. Orphanet J. Rare Dis. *8*, 49.

19. Van den Hout, J.M., Reuser, A.J., de Klerk, J.B., Arts, W.F., Smeitink, J.A., and Van der Ploeg, A.T. (2001). Enzyme therapy for pompe disease with recombinant human alpha-glucosidase from rabbit milk. J. Inherit. Metab. Dis. *24*, 266–274.

20. Kishnani, P.S., Corzo, D., Leslie, N.D., Gruskin, D., Van der Ploeg, A., Clancy, J.P., Parini, R., Morin, G., Beck, M., Bauer, M.S., et al. (2009). Early treatment with alglucosidase alpha prolongs long-term survival of infants with Pompe disease. Pediatr. Res. *66*, 329–335.

21. Strothotte, S., Strigl-Pill, N., Grunert, B., Kornblum, C., Eger, K., Wessig, C., Deschauer, M., Breunig, F., Glocker, F.X., Vielhaber, S., et al. (2010). Enzyme replacement therapy with alglucosidase alfa in 44 patients with late-onset glycogen storage disease type 2: 12-month results of an observational clinical trial. J. Neurol. *257*, 91–97.

22. Angelini, C., Semplicini, C., Ravaglia, S., Bembi, B., Servidei, S., Pegoraro, E., Moggio, M., Filosto, M., Sette, E., Crescimanno, G., et al. (2012). Observational clinical study in juvenile-adult glycogenosis type 2 patients undergoing enzyme replacement therapy for up to 4 years. J. Neurol. *259*, 952–958.

23. Harlaar, L., Hogrel, J.Y., Perniconi, B., Kruijshaar, M.E., Rizopoulos, D., Taouagh, N., Canal, A., Brusse, E., van Doorn, P.A., van der Ploeg, A.T., et al. (2019). Large variation in effects during 10 years of enzyme therapy in adults with Pompe disease. Neurology *93*, e1756–e1767.

24. Kuperus, E., Kruijshaar, M.E., Wens, S.C.A., de Vries, J.M., Favejee, M.M., van der Meijden, J.C., Rizopoulos, D., Brusse, E., van Doorn, P.A., van der Ploeg, A.T., et al. (2017). Long-term benefit of enzyme replacement therapy in Pompe disease: a 5-year prospective study. Neurology *89*, 2365–2373.

25. Chakrapani, A., Vellodi, A., Robinson, P., Jones, S., and Wraith, J.E. (2010). Treatment of infantile Pompe disease with alglucosidase alpha: the UK experience. J. Inherit. Metab. Dis. *33*, 747–750.

26. Stepien, K.M., Hendriksz, C.J., Roberts, M., and Sharma, R. (2016). Observational clinical study of 22 adult-onset Pompe disease patients undergoing enzyme replacement therapy over 5years. Mol. Genet. Metab. *117*, 413–418.

27. Kishnani, P.S., Corzo, D., Nicolino, M., Byrne, B., Mandel, H., Hwu, W.L., Leslie, N., Levine, J., Spencer, C., McDonald, M., et al. (2007). Recombinant human acid [alpha]-glucosidase: major clinical benefits in infantile-onset Pompe disease. Neurology *68*, 99–109.

28. Anderson, L.J., Henley, W., Wyatt, K.M., Nikolaou, V., Waldek, S., Hughes, D.A., Lachmann, R.H., and Logan, S. (2014). Effectiveness of enzyme replacement therapy in adults with late-onset Pompe disease: results from the NCS-LSD cohort study. J. Inherit. Metab. Dis. *37*, 945–952.

29. van der Ploeg, A.T., Clemens, P.R., Corzo, D., Escolar, D.M., Florence, J., Groeneveld, G.J., Herson, S., Kishnani, P.S., Laforet, P., Lake, S.L., et al. (2010). A randomized study of alglucosidase alfa in late-onset Pompe's disease. N. Engl. J. Med. *362*, 1396–1406.

30. Ebbink, B.J., Poelman, E., Aarsen, F.K., Plug, I., Regal, L., Muentjes, C., van der Beek, N., Lequin, M.H., van der Ploeg, A.T., and van den Hout, J.M.P. (2018). Classic infantile Pompe patients approaching adulthood: a cohort study on consequences for the brain. Dev. Med. Child Neurol. *60*, 579–586.

31. DeRuisseau, L.R., Fuller, D.D., Qiu, K., DeRuisseau, K.C., Donnelly, W.H., Jr., Mah, C., Reier, P.J., and Byrne, B.J. (2009). Neural deficits contribute to respiratory insufficiency in Pompe disease. Proc. Natl. Acad. Sci. U S A *106*, 9419–9424.

32. Pena, L.D., Proia, A.D., and Kishnani, P.S. (2015). Postmortem findings and clinical correlates in individuals with infantile-onset pompe disease. JIMD Rep. *23*, 45–54.

33. Brooks, D.A., Kakavanos, R., and Hopwood, J.J. (2003). Significance of immune response to enzyme-replacement therapy for patients with a lysosomal storage disorder. Trends Mol. Med. *9*, 450–453.

34. Wang, J., Lozier, J., Johnson, G., Kirshner, S., Verthelyi, D., Pariser, A., Shores, E., and Rosenberg, A. (2008). Neutralizing antibodies to therapeutic enzymes: considerations for testing, prevention and treatment. Nat. Biotechnol. *26*, 901–908.

35. Banugaria, S.G., Prater, S.N., Ng, Y.K., Kobori, J.A., Finkel, R.S., Ladda, R.L., Chen, Y.T., Rosenberg, A.S., and Kishnani, P.S. (2011). The impact of antibodies on clinical outcomes in diseases treated with therapeutic protein: lessons learned from infantile Pompe disease. Genet. Med. *13*, 729–736.

36. van Gelder, C.M., Hoogeveen-Westerveld, M., Kroos, M.A., Plug, I., van der Ploeg, A.T., and Reuser, A.J. (2015). Enzyme therapy and immune response in relation to CRIM status: the Dutch experience in classic infantile Pompe disease. J. Inherit. Metab. Dis. *38*, 305–314.

37. Kishnani, P.S., Goldenberg, P.C., DeArmey, S.L., Heller, J., Benjamin, D., Young, S., Bali, D., Smith, S.A., Li, J.S., Mandel, H., et al. (2010). Cross-reactive immunologic material status affects treatment outcomes in Pompe disease infants. Mol. Genet. Metab. *99*, 26–33.

38. Berrier, K.L., Kazi, Z.B., Prater, S.N., Bali, D.S., Goldstein, J., Stefanescu, M.C., Rehder, C.W., Botha, E.G., Ellaway, C., Bhattacharya, K., et al. (2015). CRIM-negative infantile Pompe disease: characterization of immune responses in patients treated with ERT monotherapy. Genet. Med. *17*, 912–918.

39. de Vries, J.M., Kuperus, E., Hoogeveen-Westerveld, M., Kroos, M.A., Wens, S.C., Stok, M., van der Beek, N.A., Kruijshaar, M.E., Rizopoulos, D., van Doorn, P.A., et al. (2017). Pompe disease in adulthood: effects of antibody formation on enzyme replacement therapy. Genet. Med. *19*, 90–97.

40. de Vries, J.M., van der Beek, N.A., Kroos, M.A., Ozkan, L., van Doorn, P.A., Richards, S.M., Sung, C.C., Brugma, J.D., Zandbergen, A.A., van der Ploeg, A.T., et al. (2010). High antibody titer in an adult with Pompe disease affects treatment with alglucosidase alfa. Mol. Genet. Metab. *101*, 338–345.

41. Desai, A.K., Kazi, Z.B., Bali, D.S., and Kishnani, P.S. (2019). Characterization of immune response in Cross-Reactive Immunological Material (CRIM)-positive infantile Pompe disease patients treated with enzyme replacement therapy. Mol. Genet. Metab. Rep. *20*, 100475.

42. Mendelsohn, N.J., Messinger, Y.H., Rosenberg, A.S., and Kishnani, P.S. (2009). Elimination of antibodies to recombinant enzyme in Pompe's disease. N. Engl. J. Med. *360*, 194–195.

43. Messinger, Y.H., Mendelsohn, N.J., Rhead, W., Dimmock, D., Hershkovitz, E., Champion, M., Jones, S.A., Olson, R., White, A., Wells, C., et al. (2012). Successful immune tolerance induction to enzyme replacement therapy in CRIM-negative infantile Pompe disease. Genet. Med. *14*, 135–142.

44. Poelman, E., Hoogeveen-Westerveld, M., Kroos-de Haan, M.A., van den Hout, J.M.P., Bronsema, K.J., van de Merbel, N.C., van der Ploeg, A.T., and Pijnappel, W. (2018). High sustained antibody titers in patients with classic infantile pompe disease following immunomodulation at start of enzyme replacement therapy. J. Pediatr. *195*, 236–243.e233.

45. Banugaria, S.G., Prater, S.N., Patel, T.T., Dearmey, S.M., Milleson, C., Sheets, K.B., Bali, D.S., Rehder, C.W., Raiman, J.A., Wang, R.A., et al. (2013). Algorithm for the early diagnosis and treatment of patients with cross reactive immunologic material-negative classic infantile pompe disease: a step towards improving the efficacy of ERT. PLoS One *8*, e67052.

46. Stenger, E.O., Kazi, Z., Lisi, E., Gambello, M.J., and Kishnani, P. (2015). Immune tolerance strategies in siblings with infantile pompe disease-advantages for a preemptive approach to high-sustained antibody titers. Mol. Genet. Metab. Rep. *4*, 30–34.

47. Elder, M.E., Nayak, S., Collins, S.W., Lawson, L.A., Kelley, J.S., Herzog, R.W., Modica, R.F., Lew, J., Lawrence, R.M., and Byrne, B.J. (2013). B-Cell depletion and immunomodulation before initiation of enzyme replacement therapy blocks the immune

response to acid alpha-glucosidase in infantile-onset Pompe disease. J. Pediatr. *163*, 847–854.e841.

48. Poelman, E., Hoogeveen-Westerveld, M., van den Hout, J.M.P., Bredius, R.G.M., Lankester, A.C., Driessen, G.J.A., Kamphuis, S.S.M., Pijnappel, W.W.M., and van der Ploeg, A.T. (2019). Effects of immunomodulation in classic infantile Pompe patients with high antibody titers. Orphanet J. Rare Dis. *14*, 71.

49. Poelman, E., van den Dorpel, J.J.A., Hoogeveen-Westerveld, M., van den Hout, J.M.P., van der Giessen, L.J., van der Beek, N., Pijnappel, W., and van der Ploeg, A.T. (2020). Effects of higher and more frequent dosing of alglucosidase alfa and immunomodulation on long-term clinical outcome of classic infantile Pompe patients. J. Inherit. Metab. Dis. *43*, 1243–1253.

50. Ferrua, F., Cicalese, M.P., Galimberti, S., Giannelli, S., Dionisio, F., Barzaghi, F., Migliavacca, M., Bernardo, M.E., Calbi, V., Assanelli, A.A., et al. (2019). Lentiviral haemopoietic stem/progenitor cell gene therapy for treatment of Wiskott-Aldrich syndrome: interim results of a non-randomised, open-label, phase 1/2 clinical study. Lancet Haematol. *6*, e239–e253.

51. Thompson, A.A., Walters, M.C., Kwiatkowski, J., Rasko, J.E.J., Ribeil, J.A., Hongeng, S., Magrin, E., Schiller, G.J., Payen, E., Semeraro, M., et al. (2018). Gene therapy in patients with transfusion-dependent beta-thalassemia. N. Engl. J. Med. *378*, 1479–1493.

52. Eichler, F., Duncan, C., Musolino, P.L., Orchard, P.J., De Oliveira, S., Thrasher, A.J., Armant, M., Dansereau, C., Lund, T.C., Miller, W.P., et al. (2017). Hematopoietic stem-cell gene therapy for cerebral adrenoleukodystrophy. N. Engl. J. Med. *377*, 1630–1638.

53. Sessa, M., Lorioli, L., Fumagalli, F., Acquati, S., Redaelli, D., Baldoli, C., Canale, S., Lopez, I.D., Morena, F., Calabria, A., et al. (2016). Lentiviral haemopoietic stem-cell gene therapy in early-onset metachromatic leukodystrophy: an ad-hoc analysis of a non-randomised, open-label, phase 1/2 trial. Lancet *388*, 476–487.

54. Hacein-Bey-Abina, S., Von Kalle, C., Schmidt, M., McCormack, M.P., Wulffraat, N., Leboulch, P., Lim, A., Osborne, C.S., Pawliuk, R., Morillon, E., et al. (2003). LMO2-associated clonal T cell proliferation in two patients after gene therapy for SCID-X1. Science *302*, 415–419.

55. Hacein-Bey-Abina, S., Garrigue, A., Wang, G.P., Soulier, J., Lim, A., Morillon, E., Clappier, E., Caccavelli, L., Delabesse, E., Beldjord, K., et al. (2008). Insertional oncogenesis in 4 patients after retrovirus-mediated gene therapy of SCID-X1. J. Clin. Invest. *118*, 3132–3142.

56. Bijvoet, A.G., van de Kamp, E.H., Kroos, M.A., Ding, J.H., Yang, B.Z., Visser, P., Bakker, C.E., Verbeet, M.P., Oostra, B.A., Reuser, A.J., et al. (1998). Generalized glycogen storage and cardiomegaly in a knockout mouse model of Pompe disease. Hum. Mol. Genet. *7*, 53–62.

57. Bijvoet, A.G., Van Hirtum, H., Vermey, M., Van Leenen, D., Van Der Ploeg, A.T., Mooi, W.J., and Reuser, A.J. (1999). Pathological features of glycogen storage disease type II highlighted in the knockout mouse model. J. Pathol. *189*, 416–424.

58. Kamphoven, J.H., Stubenitsky, R., Reuser, A.J., Van Der Ploeg, A.T., Verdouw, P.D., and Duncker, D.J. (2001). Cardiac remodeling and contractile function in acid alpha-glucosidase knockout mice. Physiol. Genomics *5*, 171–179.

59. van Til, N.P., Stok, M., Aerts Kaya, F.S., de Waard, M.C., Farahbakhshian, E., Visser, T.P., Kroos, M.A., Jacobs, E.H., Willart, M.A., et al. (2010). Lentiviral gene therapy of murine hematopoietic stem cells ameliorates the Pompe disease phenotype. Blood *115*, 5329–5337.

60. Stok, M., de Boer, H., Huston, M.W., Jacobs, E.H., Roovers, O., Visser, T.P., Jahr, H., Duncker, D.J., van Deel, E.D., Reuser, A.J.J., et al. (2020). Lentiviral hematopoietic stem cell gene therapy corrects murine pompe disease. Mol. Ther. - Methods Clin. Dev. *17*, 1014–1025.

61. Raben, N., Danon, M., Gilbert, A.L., Dwivedi, S., Collins, B., Thurberg, B.L., Mattaliano, R.J., Nagaraju, K., and Plotz, P.H. (2003). Enzyme replacement therapy in the mouse model of Pompe disease. Mol. Genet. Metab. *80*, 159–169.

62. Douillard-Guilloux, G., Richard, E., Batista, L., and Caillaud, C. (2009). Partial phenotypic correction and immune tolerance induction to enzyme replacement therapy after hematopoietic stem cell gene transfer of alpha-glucosidase in Pompe disease. J. Gene Med. *11*, 279–287.

63. Sun, B., Bird, A., Young, S.P., Kishnani, P.S., Chen, Y.T., and Koeberl, D.D. (2007). Enhanced response to enzyme replacement therapy in Pompe disease after the induction of immune tolerance. Am. J. Hum. Genet. *81*, 1042–1049.

64. Leshem, B., Tsuberi, B.Z., Lebendiker, Z., Anafi-Ayalon, M., Shalit, M., Weiss, L., Slavin, S., and Kedar, E. (1987). Bone marrow transplantation with T-cell-depleted grafts. II. Reconstitution of immunohemopoietic functions in lethally irradiated mice transplanted with unseparated or T-cell-depleted bone marrow grafts disparate at minor histocompatibility antigens. Transplantation *43*, 814–817.

65. Filip, S., Mokry, J., Vavrova, J., Sinkorova, Z., Micuda, S., Sponer, P., Filipova, A., Hrebikova, H., and Dayanithi, G. (2014). The peripheral chimerism of bone marrow-derived stem cells after transplantation: regeneration of gastrointestinal tissues in lethally irradiated mice. J. Cell Mol. Med. *18*, 832–843.

66. van Gelder, C.M., Poelman, E., Plug, I., Hoogeveen-Westerveld, M., van der Beek, N.A., Reuser, A.J., and van der Ploeg, A.T. (2016). Effects of a higher dose of alglucosidase alfa on ventilator-free survival and motor outcome in classic infantile Pompe disease: an open-label single-center study. J. Inherit. Metab. Dis. *39*, 383–390.

67. Sun, B., Li, S., Bird, A., Yi, H., Kemper, A., Thurberg, B.L., and Koeberl, D.D. (2010). Antibody formation and mannose-6-phosphate receptor expression impact the efficacy of muscle-specific transgene expression in murine Pompe disease. J. Gene Med. *12*, 881–891.

68. Ponder, K.P. (2008). Immune response hinders therapy for lysosomal storage diseases. J. Clin. Invest. *118*, 2686–2689.

69. Koeberl, D.D., and Kishnani, P.S. (2009). Immunomodulatory gene therapy in lysosomal storage disorders. Curr. Gene Ther. *9*, 503–510.

70. Saif, M.A., Bigger, B.W., Brookes, K.E., Mercer, J., Tylee, K.L., Church, H.J., Bonney, D.K., Jones, S., Wraith, J.E., and Wynn, R.F. (2012). Hematopoietic stem cell transplantation improves the high incidence of neutralizing allo-antibodies observed in Hurler's syndrome after pharmacological enzyme replacement therapy. Haematologica *97*, 1320–1328.

71. Masat, E., Laforêt, P., De Antonio, M., Corre, G., Perniconi, B., Taouagh, N., Mariampillai, K., Amelin, D., Mauhin, W., Hogrel, J.Y., et al. (2016). Long-term exposure to Myozyme results in a decrease of anti-drug antibodies in late-onset Pompe disease patients. Sci. Rep. *6*, 36182.

72. Ghosh, A., Miller, W., Orchard, P.J., Jones, S.A., Mercer, J., Church, H.J., Tylee, K., Lund, T., Bigger, B.W., Tolar, J., et al. (2016). Enzyme replacement therapy prior to haematopoietic stem cell transplantation in Mucopolysaccharidosis Type I: 10 year combined experience of 2 centres. Mol. Genet. Metab. *117*, 373–377.

73. Murphy, K.M., Heimberger, A.B., and Loh, D.Y. (1990). Induction by antigen of intrathymic apoptosis of CD4+CD8+TCRlo thymocytes in vivo. Science *250*, 1720–1723.

74. Liblau, R.S., Tisch, R., Shokat, K., Yang, X., Dumont, N., Goodnow, C.C., and McDevitt, H.O. (1996). Intravenous injection of soluble antigen induces thymic and peripheral T-cells apoptosis. Proc. Natl. Acad. Sci. U S A *93*, 3031–3036.

75. Chen, Y., Luo, X., Schroeder, J.A., Chen, J., Baumgartner, C.K., Hu, J., and Shi, Q. (2017). Immune tolerance induced by platelet-targeted factor VIII gene therapy in hemophilia A mice is CD4 T cell mediated. J. Thromb. Haemost. *15*, 1994–2004.

76. Squeri, G., Passerini, L., Ferro, F., Laudisa, C., Tomasoni, D., Deodato, F., Donati, M.A., Gasperini, S., Aiuti, A., Bernardo, M.E., et al. (2019). Targeting a pre-existing anti-transgene T cell response for effective gene therapy of MPS-I in the mouse model of the disease. Mol. Ther. *27*, 1215–1227.

77. Garcia-Perez, L., van Roon, L., Schilham, M.W., Lankester, A.C., Pike-Overzet, K., and Staal, F.J.T. (2021). Combining mobilizing agents with busulfan to reduce chemotherapy-based conditioning for hematopoietic stem cell transplantation. Cells *10*, 1077.

78. Uchida, N., Nassehi, T., Drysdale, C.M., Gamer, J., Yapundich, M., Bonifacino, A.C., Krouse, A.E., Linde, N., Hsieh, M.M., Donahue, R.E., et al. (2019). Busulfan combined with immunosuppression allows efficient engraftment of gene-modified cells in a rhesus macaque model. Mol. Ther. *27*, 1586–1596.

79. Fontanellas, A., Hervas-Stubbs, S., Mauleon, I., Dubrot, J., Mancheno, U., Collantes, M., Sampedro, A., Unzu, C., Alfaro, C., Palazon, A., et al. (2010). Intensive pharmacological immunosuppression allows for repetitive liver gene transfer with recombinant adenovirus in nonhuman primates. Mol. Ther. *18*, 754–765.

80. Lankester, A.C., Albert, M.H., Booth, C., Gennery, A.R., Güngör, T., Hönig, M., Morris, E.C., Moshous, D., Neven, B., Schulz, A., et al. (2021). EBMT/ESID inborn errors working party guidelines for hematopoietic stem cell transplantation for inborn errors of immunity. Bone Marrow Transpl. *56*, 2052–2062.

81. Spada, M., Pagliardini, V., Ricci, F., Biamino, E., Mongini, T., and Porta, F. (2018). Early higher dosage of alglucosidase alpha in classic Pompe disease. J. Pediatr. Endocrinol. Metab. *31*, 1343–1347.

82. Parini, R., De Lorenzo, P., Dardis, A., Burlina, A., Cassio, A., Cavarzere, P., Concolino, D., Della Casa, R., Deodato, F., Donati, M.A., et al. (2018). Long term clinical history of an Italian cohort of infantile onset Pompe disease treated with enzyme replacement therapy. Orphanet J. Rare Dis. *13*, 32.

83. Nossal, G.J. (1994). Negative selection of lymphocytes. Cell *76*, 229–239.

84. Bagley, J., Tian, C., Sachs, D.H., and Iacomini, J. (2002). Induction of T-cell tolerance to an MHC class I alloantigen by gene therapy. Blood *99*, 4394–4399.

85. Kang, E.S., and Iacomini, J. (2002). Induction of central deletional T cell tolerance by gene therapy. J. Immunol. *169*, 1930–1935.

86. Krenger, W., Blazar, B.R., and Hollander, G.A. (2011). Thymic T-cell development in allogeneic stem cell transplantation. Blood *117*, 6768–6776.

87. Forman, D., Kang, E.S., Tian, C., Paez-Cortez, J., and Iacomini, J. (2006). Induction of alloreactive CD4 T cell tolerance in molecular chimeras: a possible role for regulatory T cells. J. Immunol. *176*, 3410–3416.

88. Zhang, P., Sun, B., Osada, T., Rodriguiz, R., Yang, X.Y., Luo, X., Kemper, A.R., Clay, T.M., and Koeberl, D.D. (2012). Immunodominant liver-specific expression suppresses transgene-directed immune responses in murine pompe disease. Hum. Gene Ther. *23*, 460–472.

89. Bond, J.E., Kishnani, P.S., and Koeberl, D.D. (2019). Immunomodulatory, liver depot gene therapy for Pompe disease. Cell Immunol. *342*, 103737.

90. Han, S.O., Ronzitti, G., Arnson, B., Leborgne, C., Li, S., Mingozzi, F., and Koeberl, D. (2017). Low-dose liver-targeted gene therapy for pompe disease enhances therapeutic efficacy of ERT via immune tolerance induction. Mol. Ther. Methods Clin. Dev. *4*, 126–136.

91. Broomfield, A., Fletcher, J., Davison, J., Finnegan, N., Fenton, M., Chikermane, A., Beesley, C., Harvey, K., Cullen, E., Stewart, C., et al. (2016). Response of 33 UK patients with infantile-onset Pompe disease to enzyme replacement therapy. J. Inherit. Metab. Dis. *39*, 261–271.

92. Kazi, Z.B., Prater, S.N., Kobori, J.A., Viskochil, D., Bailey, C., Gera, R., Stockton, D.W., McIntosh, P., Rosenberg, A.S., and Kishnani, P.S. (2016). Durable and sustained immune tolerance to ERT in Pompe disease with entrenched immune responses. JCI Insight *1*, e86821.

93. Zufferey, R., Dull, T., Mandel, R.J., Bukovsky, A., Quiroz, D., Naldini, L., and Trono, D. (1998). Self-inactivating lentivirus vector for safe and efficient in vivo gene delivery. J. Virol. *72*, 9873–9880.

94. Naldini, L., Blomer, U., Gage, F.H., Trono, D., and Verma, I.M. (1996). Efficient transfer, integration, and sustained long-term expression of the transgene in adult rat brains injected with a lentiviral vector. Proc. Natl. Acad. Sci. U S A *93*, 11382–11388.

95. Dull, T., Zufferey, R., Kelly, M., Mandel, R.J., Nguyen, M., Trono, D., and Naldini, L. (1998). A third-generation lentivirus vector with a conditional packaging system. J. Virol. *72*, 8463–8471.

96. Okumiya, T., Keulemans, J.L., Kroos, M.A., Van der Beek, N.M., Boer, M.A., Takeuchi, H., Van Diggelen, O.P., and Reuser, A.J. (2006). A new diagnostic assay for glycogen storage disease type II in mixed leukocytes. Mol. Genet. Metab. *88*, 22–28.

97. van der Wal, E., Bergsma, A.J., van Gestel, T.J.M., In 't Groen, S.L.M., Zaehres, H., Arauzo-Bravo, M.J., Scholer, H.R., van der Ploeg, A.T., and Pijnappel, W. (2017). GAA deficiency in pompe disease is alleviated by exon inclusion in iPSC-derived skeletal muscle cells. Mol. Ther. Nucleic Acids *7*, 101–115.

OMTM, Volume *25*

Supplemental information

Lentiviral gene therapy prevents anti-human acid α-glucosidase antibody formation in murine Pompe disease

Qiushi Liang, Eva C. Vlaar, Fabio Catalano, Joon M. Pijnenburg, Merel Stok, Yvette van Helsdingen, Arnold G. Vulto, Wendy W.J. Unger, Ans T. van der Ploeg, W.W.M. Pim Pijnappel, and Niek P. van Til

Table S1. Sequences of primers for qPCR

Primers	Sequence
HIV-U3 forward	5′-CTGGAAGGGCTAATTCACTC-3′
HIV-PSI reverse	5′-GGTTTCCCTTTCGCTTTCAG-3′
Sry forward	5′-TCATCGGAGGGCTAAAGTGTCAC-3′
Sry reverse	5′-TGGCATGTGGGTTCCTGTCC-3′
Gapdh forward	5′- TAATGGGGAGAGGTTCGATG -3′
Gapdh reverse	5′- GCTGCTTCCCGAGTAAAATG -3′

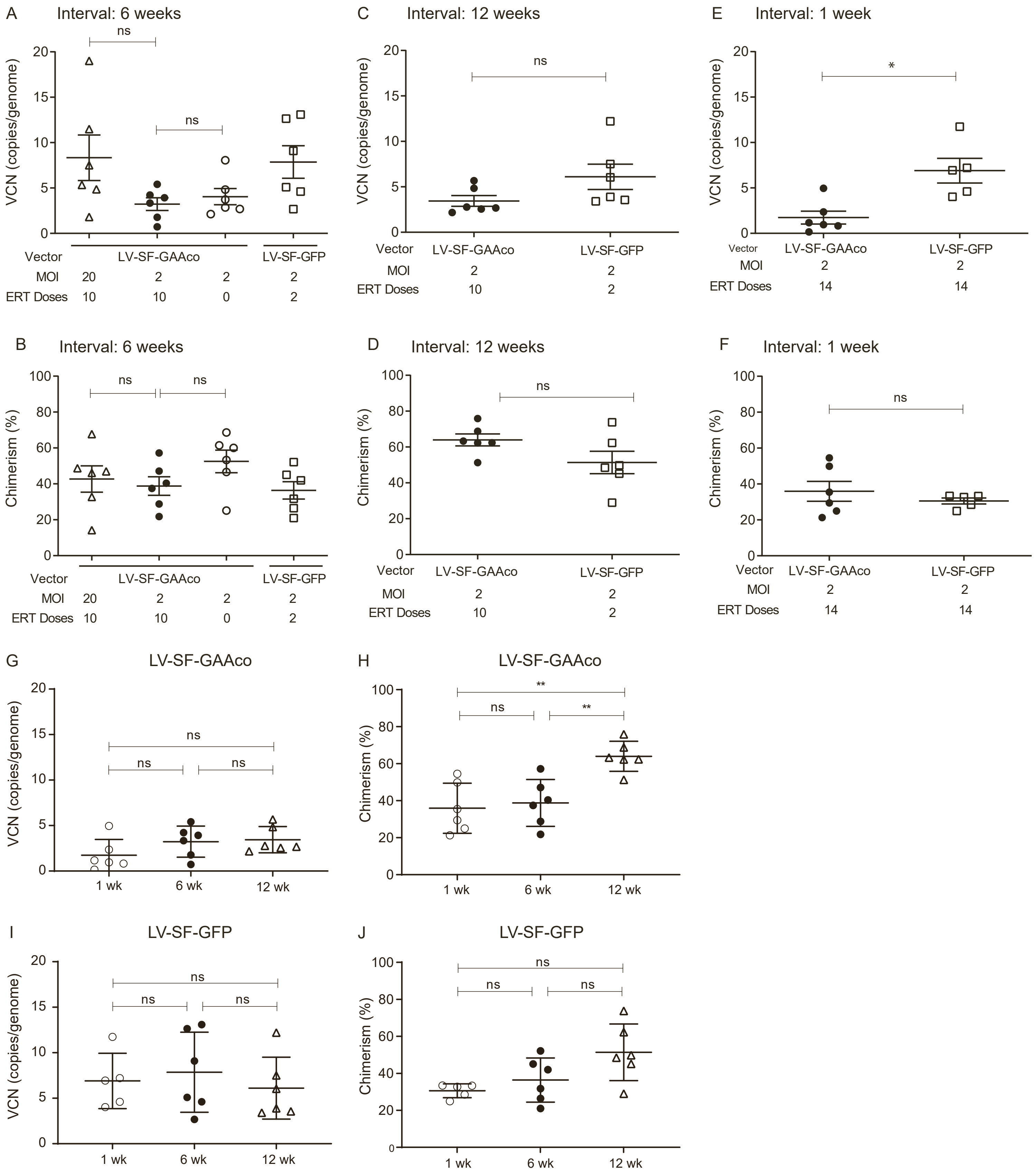

Fig. S1. Timing of immune tolerance induction: vector copy number and chimerism.

Vector copy number and chimerism in bone marrow. Graphs and symbols correspond to the experiments shown in Fig. 1. ERT was started 6 weeks (A, B), 12 weeks (C, D), or 1 week (E, F) after gene therapy. Side-by-side comparisons of vector copy number and chimerism in mice treated with LV-SF-GAA (G, H) and LV-SF-GFP (I, J). Bone marrow was collected at the end of the experiment and vector copy number and chimerism were determined by HIV and Sry qPCR, respectively, and normalized using Gapdh. Data are represented as means ± SEM; n=6 is all groups except in LV-SF-GFP treated mice in panels E and F (n=5). *p<0.05; ** p <0.01; ns, not significant.

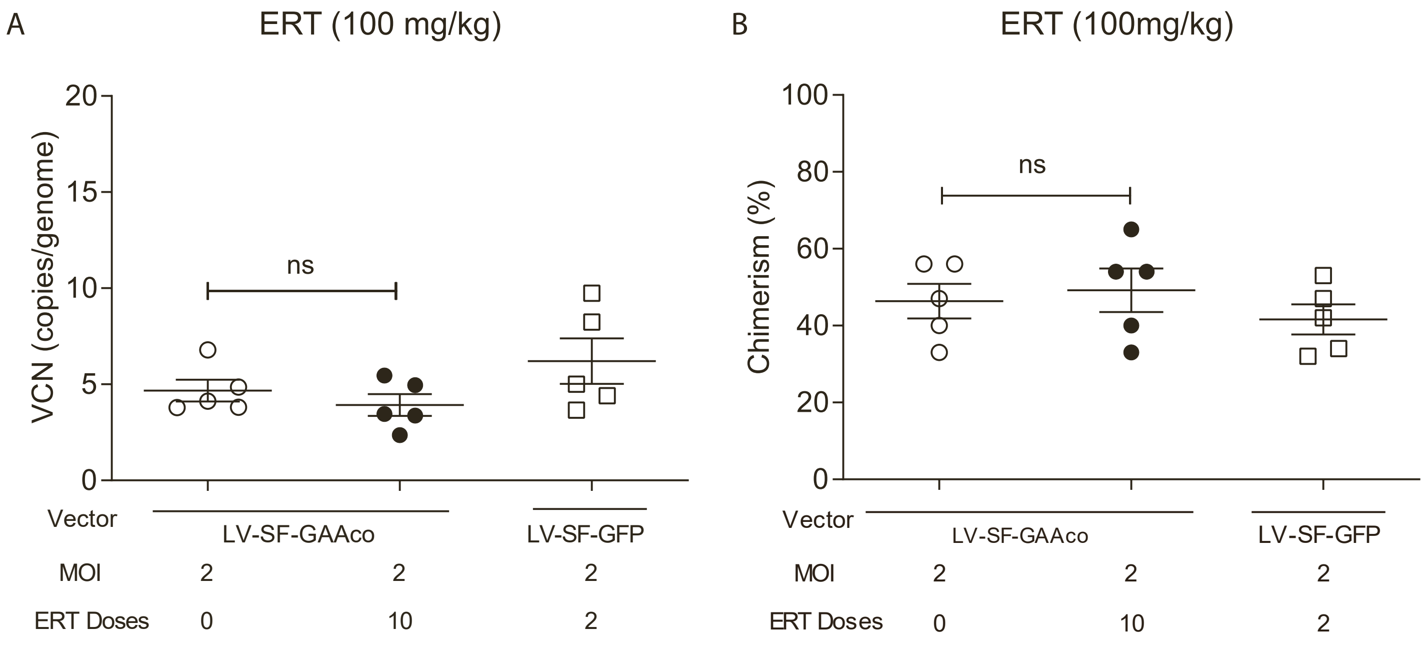

Fig. S2. Vector copy number and chimerism in mice receiving ERT doses at 100 mg/kg.
Graphs and symbols correspond to the experiment shown in Fig. 3. Vector copy number (A) and chimerism (B) were determined as described in in Fig. S1. Data are represented as means ± SEM; n=5 per group. Ns, not significant.
